# Supplementary material for: Temporo-spatial cellular atlas of the regenerating alveolar niche in idiopathic pulmonary fibrosis
Source: Nat Commun. 2025 Aug 4;16:7150. doi: 10.1038/s41467-025-61880-1 (PMC12322046; doi:10.1038/s41467-025-61880-1)
Supplement: Supplementary file 1 — Supplementary Information [file 41467_2025_61880_MOESM1_ESM.pdf]

Examine UMAP (a) and define clusters using heatmaps showing median marker expression (b), expression density histograms which allow better delineation of the range of marker expression (c). Check cluster distribution across all sections (d) (which shows the frequency of each cluster in different samples and disease stage) e.g. to ensure the cluster is not only found in one sample.

The following cells are filtered:

- No antibody staining (7.9% of total cells)
- Cluster makes up <0.1% of total cells (5 clusters)
- Merge clusters that are similar on UMAP and cannot be called another cluster - clusters 34 and 6 (ATII) and clusters 20 and 21(CD4 T cells) were merged.

Determine quality of IMC staining using expression density histogram (c) and MCD image (e) – relegate antibodies with high background, poor or diffuse staining to lower ranking antibodies in decision trees for annotation e.g. BDCA2 and KRT14

Determine which cells are epithelial, immune cells or non-epithelial structural cells , and work on annotation within these groups

Further refine cluster identities by visualising spatial location of clusters using cell centroid plots (f) and H&E stained lung section from adjacent lung sections (g),(h)

sense-check annotated clusters against MCD images (e), cell centroid plots (f) and H&E sections (g)

a. UMAP (of dataset but in 2D to complement 3D UMAP in Figure 1O)

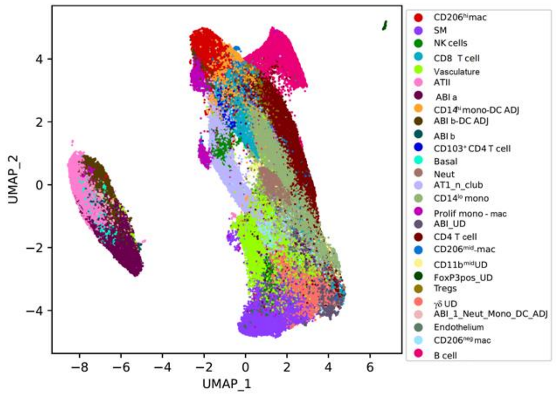

b. Heatmap showing median marker expression

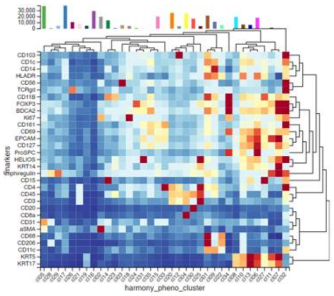

c. Marker expression density histogram (exemplar showing markers CD56 and CD68)

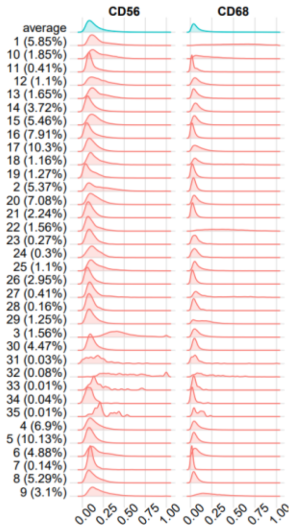

d. Cluster distribution plots

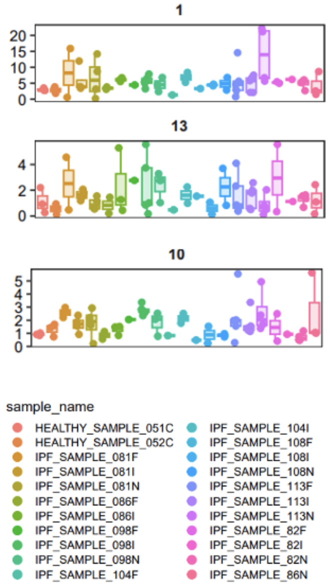

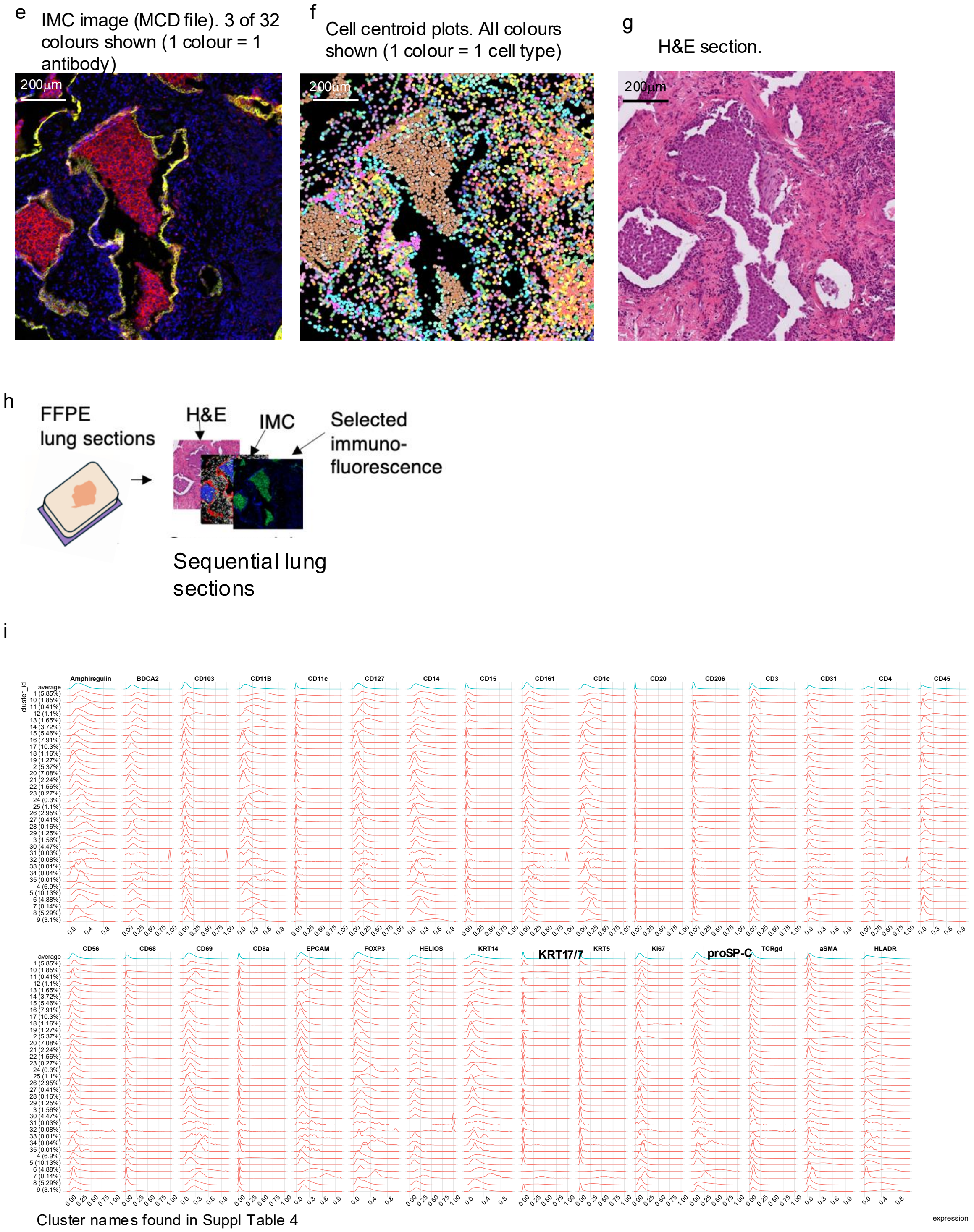

Supplementary Fig. 1. Annotation workflow. Arrows indicated iterative loops to enrich accuracy of annotation. a-h are examples for different steps of the annotation flow, mentioned (in parenthesis) in the annotation work flow. g. Full expression density plot for all 31 antibodies, excluding the two DNA probes that make up the 33-plex panel. IMC – imaging mass cytometry; H&E – haematoxylin and eosin; FFPE – formalin fixed and paraffin embedded.

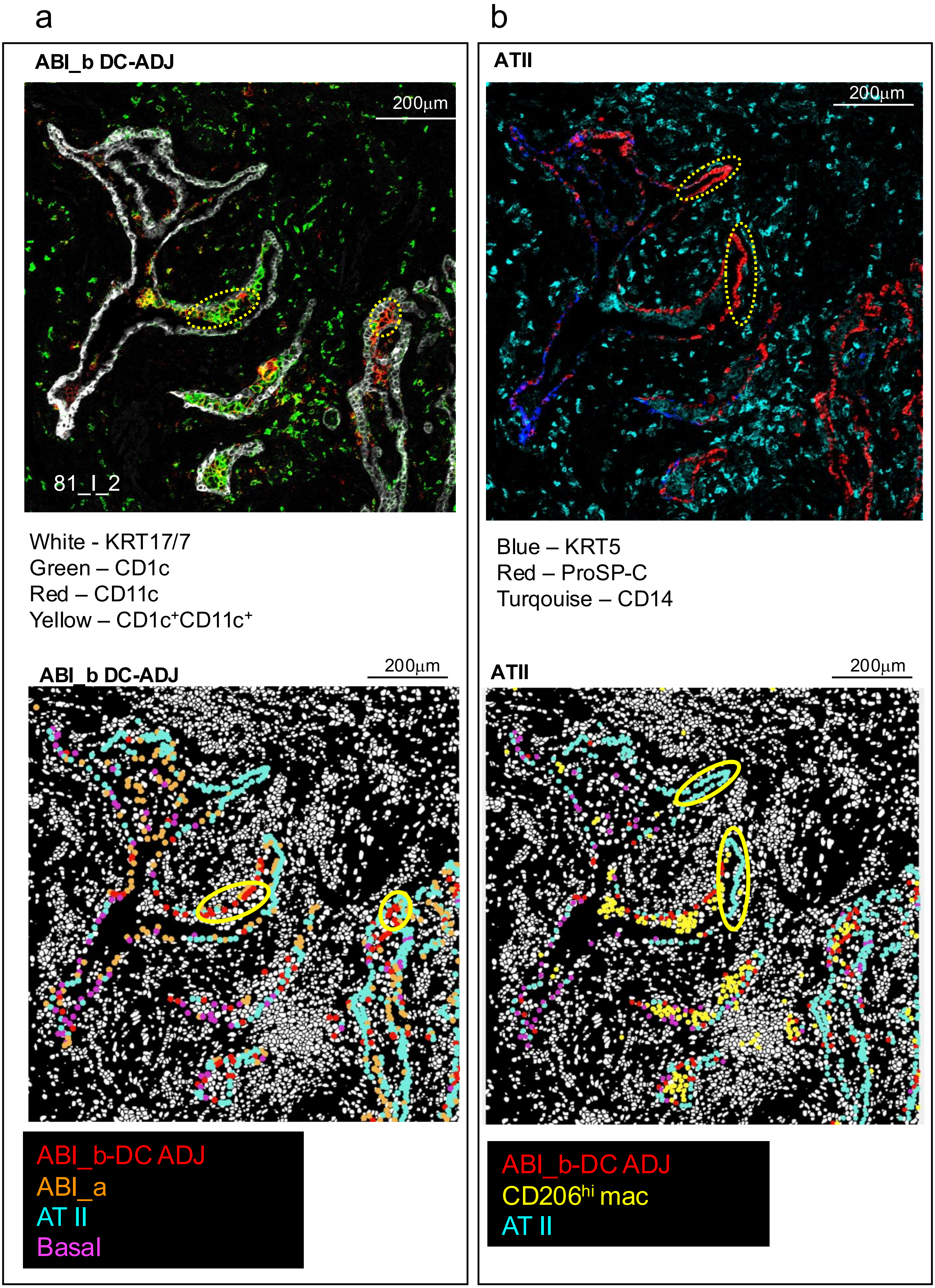
a
ABI_b DC-ADJ
200µm
81_I_2
White - KRT17/7
Green – CD1c
Red – CD11c
Yellow – CD1c+CD11c+
ABI_b DC-ADJ
200µm
ABI_b-DC ADJ
ABI_a
AT II
Basal
b
ATII
200µm
Blue – KRT5
Red – ProSP-C
Turqouise – CD14
ATII
200µm
ABI_b-DC ADJ
CD206hi mac
AT II

Supplementary Fig. 2. ABI_b-DC ADJ cell cluster and AT II cells. IMC staining (MCD file image) showing ABI_b-DC ADJ cell cluster and AT II cells **a.** Top panel – circles show presence of CD11c and CD1c (red and green respectively) beside KRT5 and KRT17/7-expressing cells (white) on IMC imaging. Bottom panel – circle show equivalent cells but now identified in cell centroid map as ABI_b-DC ADJ cell (red). **b.** Circles in top panel show $KRT5^{-}ProSpC^{+}$ ATII cells and bottom panel, the equivalent cell centroid map showing ATII cells (circled). Note that the alveolar epithelial cells in the regenerating niches in IPF lungs do not appear normal morphologically (ie single ATII spaced out in an alveolar epithelial lining). Instead they appear in continuous rows of AT II, histopathologically identifiable (on H&E staining) as type II alveolar metaplasia.

a. **Habermann AC et al Sci Adv 2020 (IPF lung tissue) (single cell transcriptomic study)**
Authors termed KRT5^{-}KRT17^{lo} cells as alveolar intermediates. There is also a KRT5^{neg} transitional AT II cell cluster.

Adams TS et al Sci Adv 20202 (IPF lung tissue) (single cell transcriptomic study)
Authors termed aberrant basaloid (AB) or KRT5^{-} basaloid cells as alveolar intermediates.

Kathiriya JJ et al Nat Cell Biol 2021(lung organoid studies) (gene expression)
See Kathriya's Fig 7a. Authors called alveolar intermediates KRT5^{neg} ABIs although there may be very low expression of KRT5 on these ABIs from their Fig 7a.

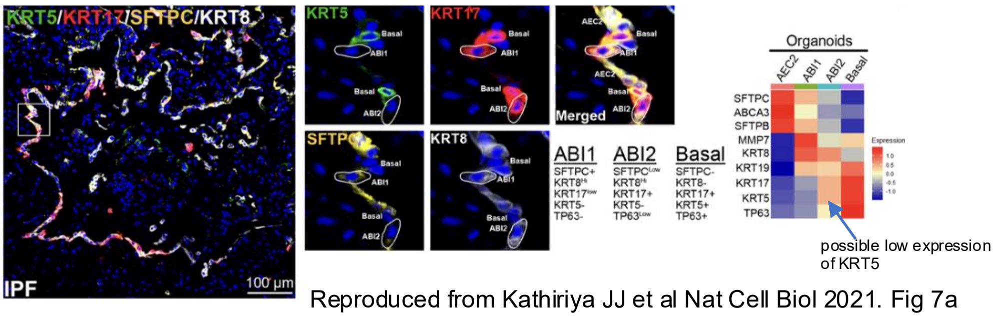

Reproduced from Kathiriya JJ et al Nat Cell Biol 2021. Fig 7a

Weeratunga P (our paper) (IPF lung tissue)(IMC, protein)
We termed alveolar intermediates:
ABI_a - KRT5^{neg-lo}proSP-C^{neg-lo}
ABI_b - KRT5^{neg-lo} proSP-C^{lo}
ABI_b - DC ADJ - KRT5^{neg-lo} proSP-C^{lo}

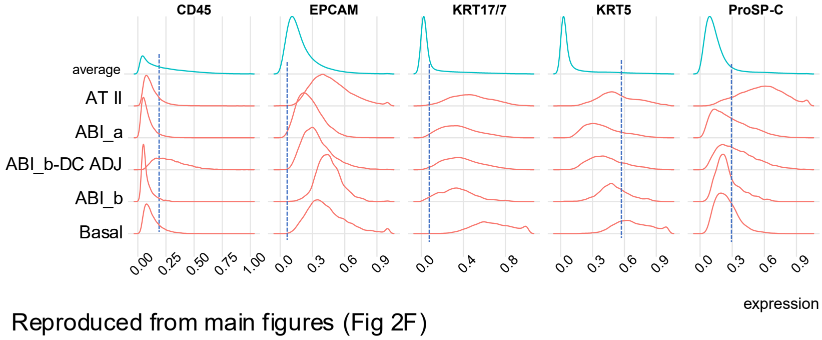

Reproduced from main figures (Fig 2F)

b. **Habermann AC et al Sci Adv 2020. Gene expression of KRT5, KRT17, KRT7 and SPFTC (proSP-C)**

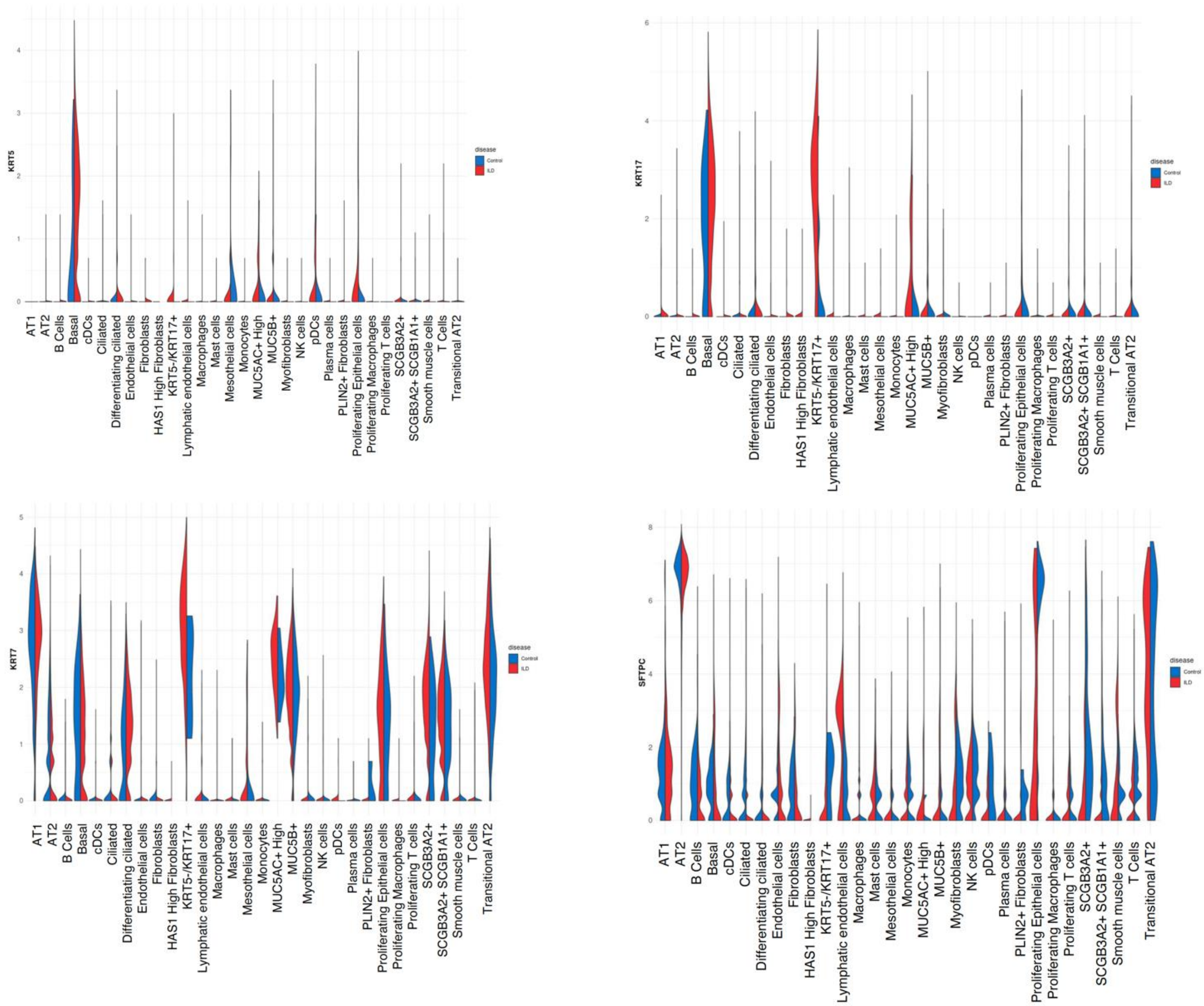

C. **Adams TS et al Sci Adv 20202**

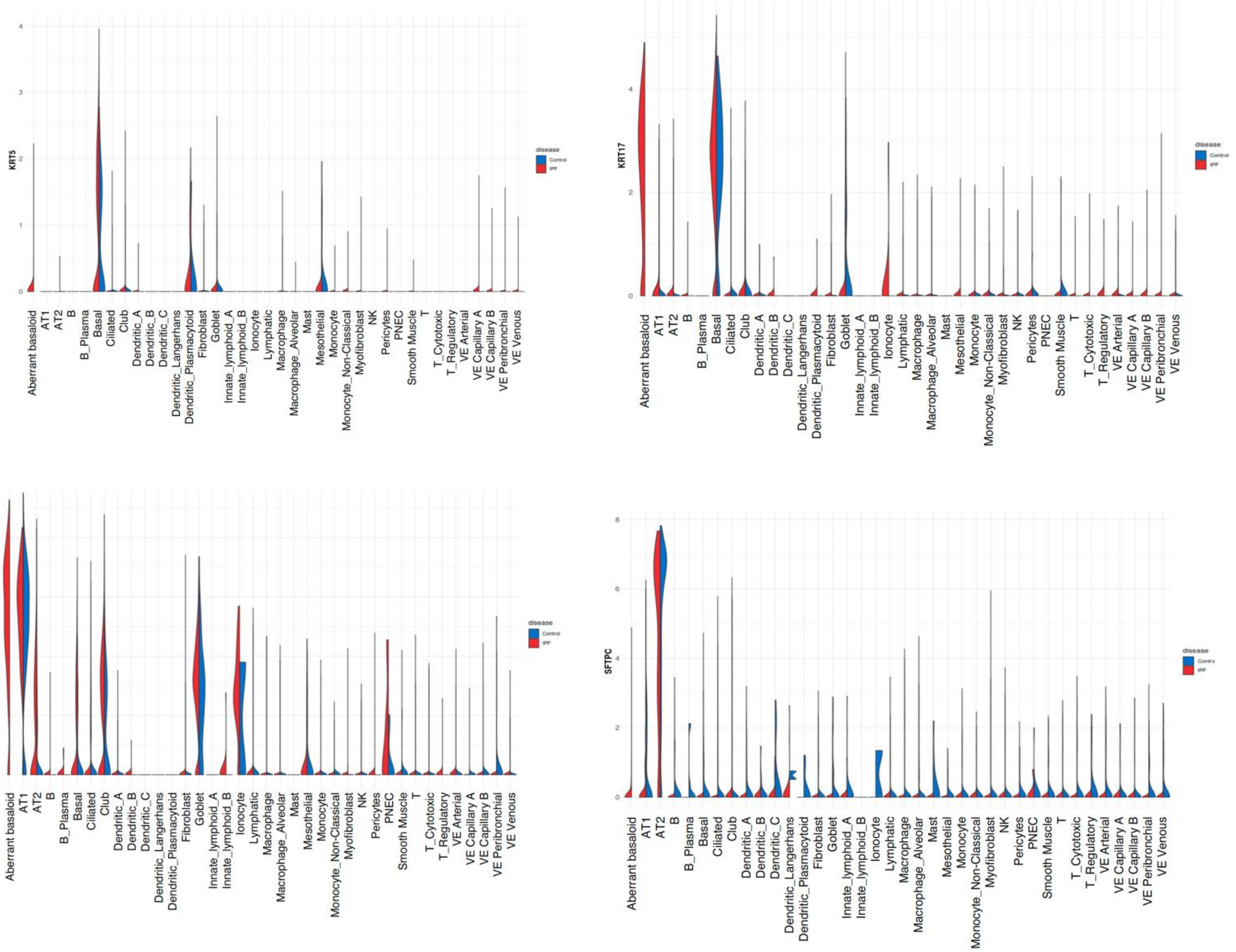

d. Combined data from Suppl Fig 3B and C shows that at gene level,

- ATI cells are $KRT5^{-}KRT17^{-}KRT7^{hi}$ proSP-C^{lo}
- ATII cells are $KRT5^{-}KRT17^{-}KRT7^{mid}$ proSP-C^{hi}
- KRT5-KRT17 AB cells are $KRT5^{-/lo}KRT17^{hi}KRT7^{hi}$ proSP-C^{lo}
- Transitional ATII cells are $KRT5^{-}KRT17^{lo}KRT7^{hi}$ proSP-C^{mid-hi}

However, note that at single cell transcriptomic level, ATI and ATII derivation from lung digest is not a complete picture due to technical difficulties of retrieving these cells, in particular, ATI cells

Proposed terms of equivalence
for our protein-led alveolar-basaloid intermediates vs Habermann, Adams and Kathiriya

ABI_a =
- Kathiriya's ABI 1 (possibly also ABI 2)
- Adam's AB
- Habermann's $KRT5^{-}KRT17^{lo}$ subset

ABI_b =
- Kathiriya's ABI 2 (possibly also ABI 1)
- Adam's AB
- Habermann's $KRT5^{-}KRT17^{lo}$ subset

e.

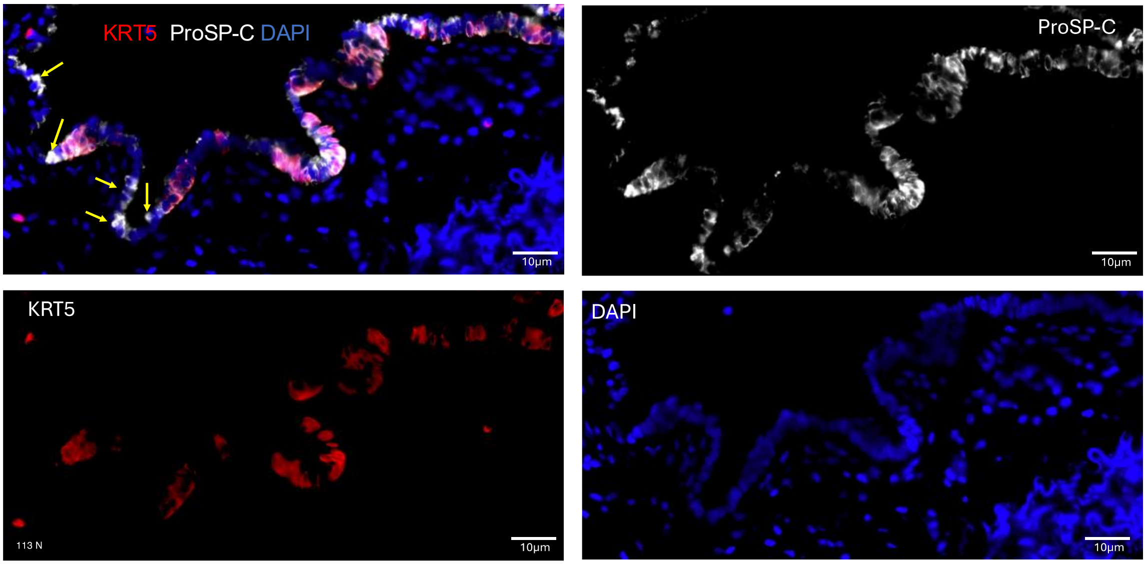

Yellow arrows – Single positive staining - ProSp-C^{+} $KRT5^{neg}$ (AT II cells). All other cells double positive for KRT5 and ProSp-C (ABI_a and ABI_b)

Weeratunga P (our paper) (IPF lung tissue)(IMC, protein)
We termed alveolar intermediates:
ABI_a - $KRT5^{neg-lo}$proSP-C^{neg-lo}
ABI_b - $KRT5^{neg-lo}$ proSP-C^{lo}
ABI_b - DC ADJ - $KRT5^{neg-lo}$ proSP-C^{lo}

f.

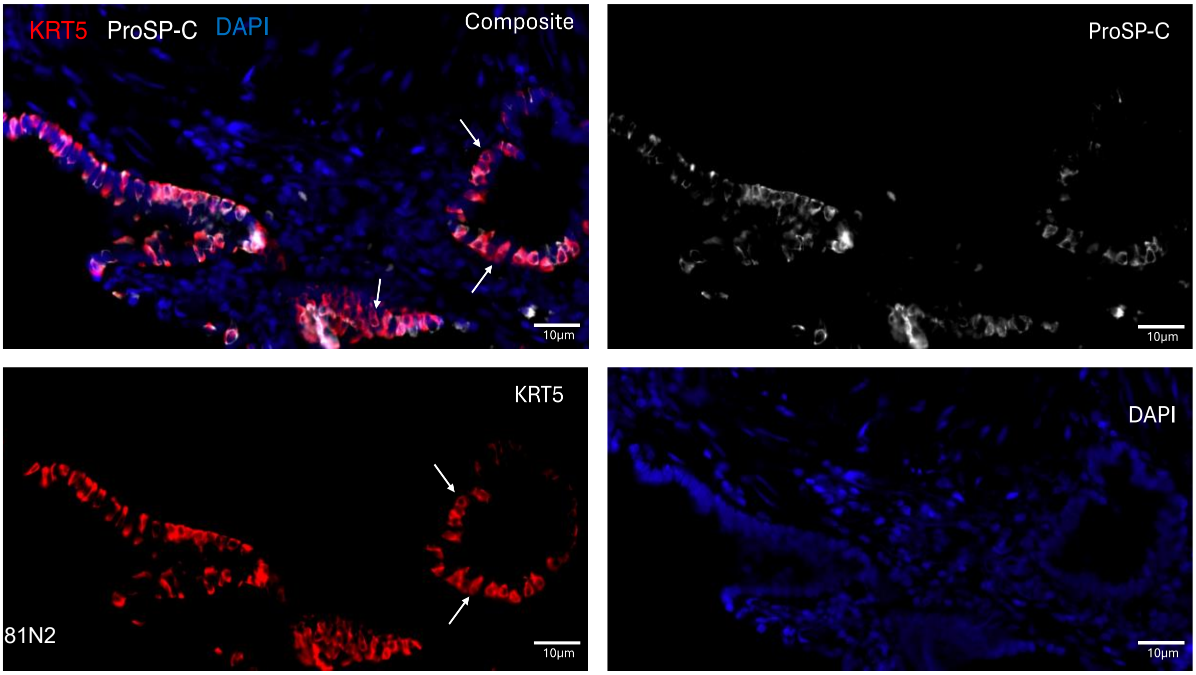

White arrows – Single positive staining - KRT5^{+} (Basal cells). All other cells double positive for KRT5 and ProSp-C (ABI_a and ABI_b)

Supplementary Fig. 3. Matching our aberrant alveolar epithelial cells to previously published findings. a. Summary of annotation terms for aberrant alveolar epithelial cells from three key papers (Habermann AC et al, Adams TS et al and Kathiriya JJ et al) [6-8] compared to our study. **b-c.** Analysis of gene expression for KRT 5, KRT 17, KRT 7 and SFPTC (ProSP-C) from publicly available single cell transcriptomic data from Habermann (**b**), Adams **(c)** showing KRT5, KRT17 and SFTPC(proSP-C) gene expression in their aberrant alveolar cell clusters (KRT5^{-}KRT17^{+} cells and AB cells respectively) compared to our protein expression in (**a**) (Weeratunga). **d.** Summary of data from (**b**) and (**c**) and proposed terms of equivalence between these data and ours. **e**. Immunofluorescence staining for proSP-C and KRT5 in IPF lung sections depicting our ABI_a, ABI_b, basal and ATII cells.

Supplementary Fig. 4

a

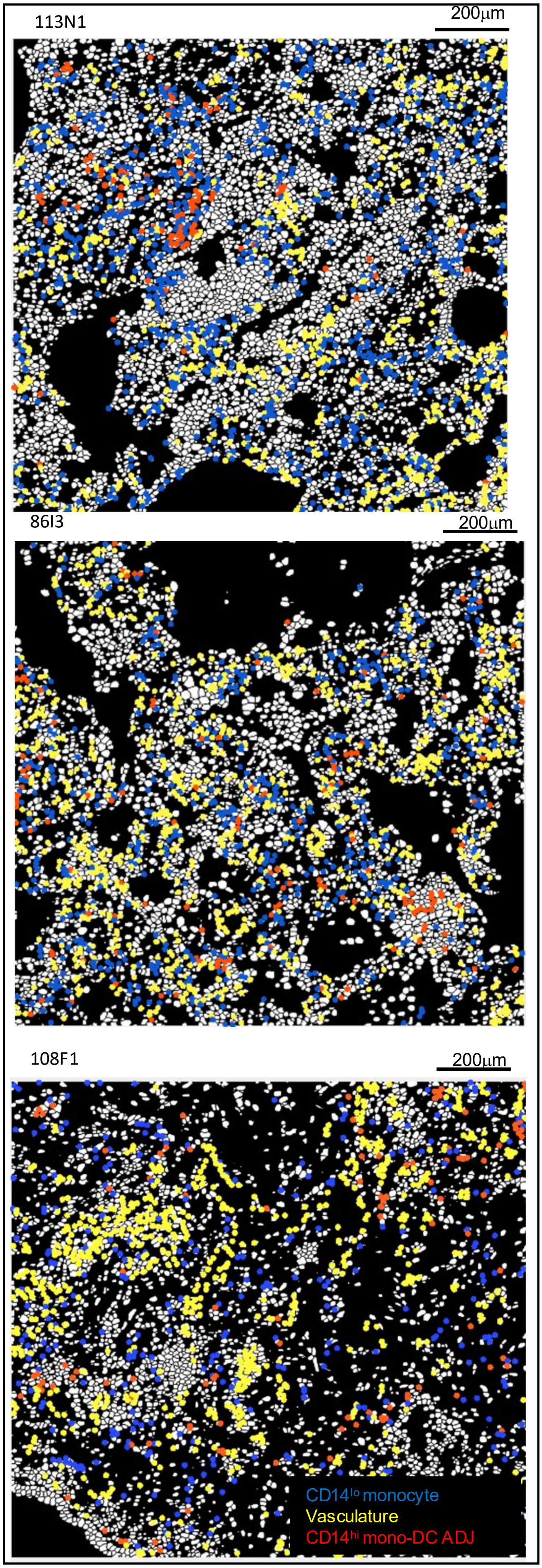

b

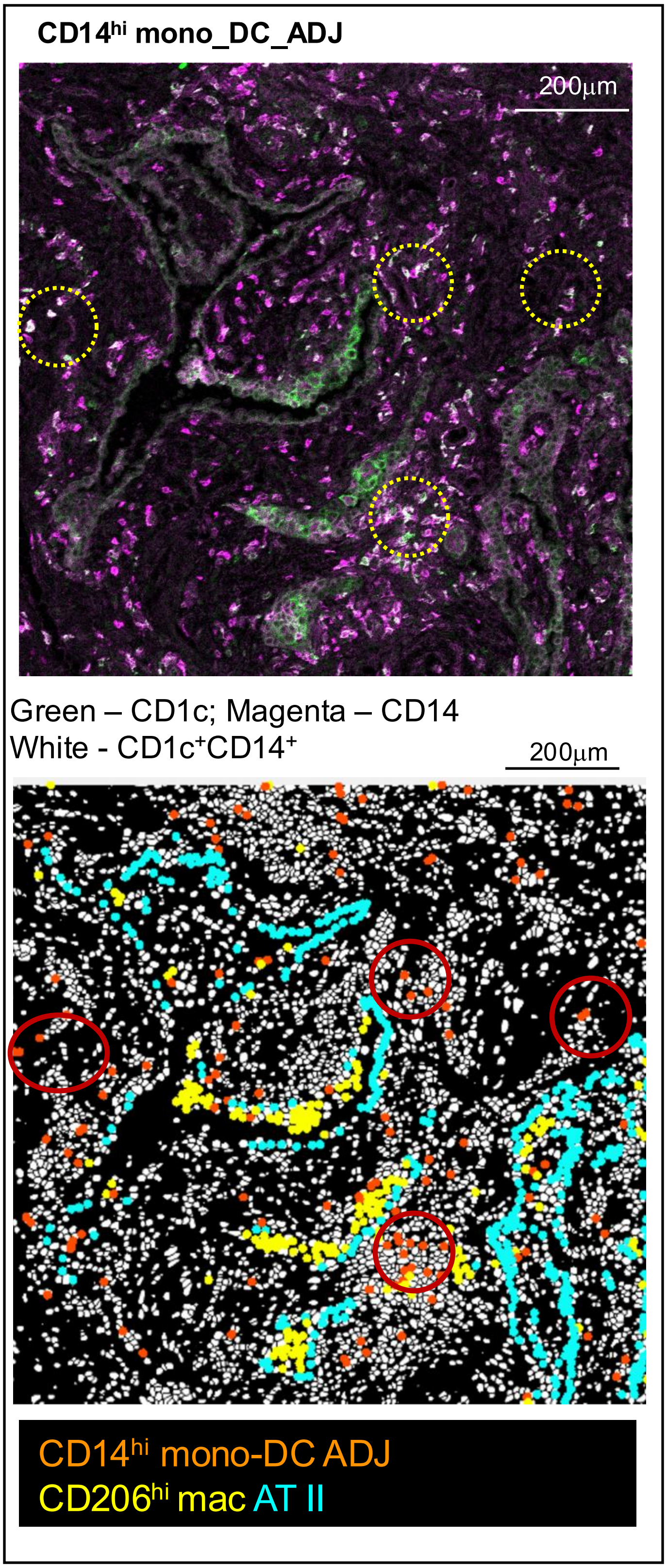

Supplementary Fig. 4 – CD14^{lo} and CD14^{hi} monocytes. **a.** Cell centroid maps showing CD14^{lo} monocytes (blue), CD14^{hi} monocytes-DC ADJ (red) and vasculature (yellow) in three different IPF lung sections (all from Intm stage). B. IMC staining (MCD file image) showing CD1c and CD14 antibody staining, and double-positive staining cells (in yellow broken circles) in the upper panel and its matching cell centroid map in the bottom panel with CD14^{hi} mono-DC ADJ cells in red circles.

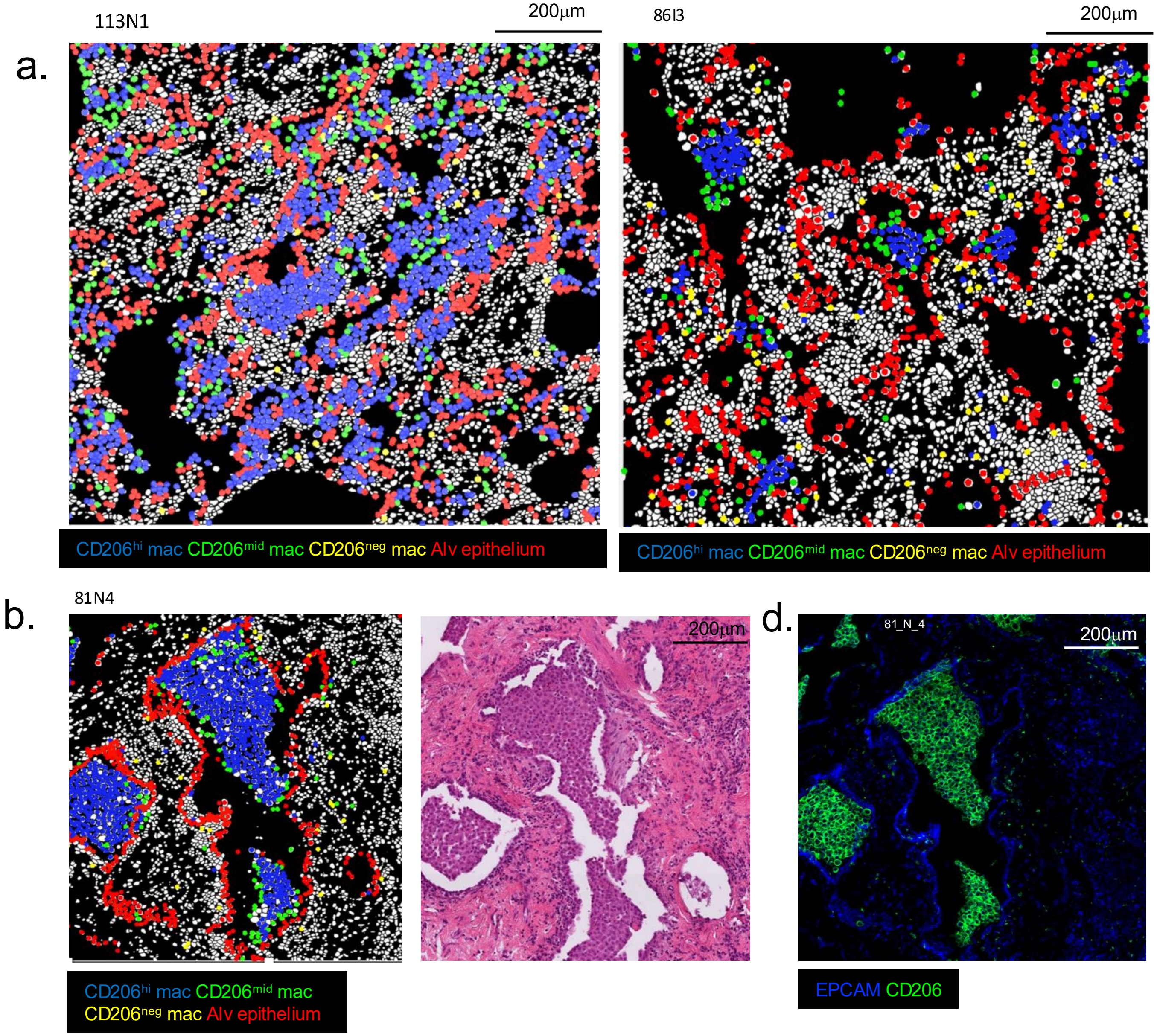

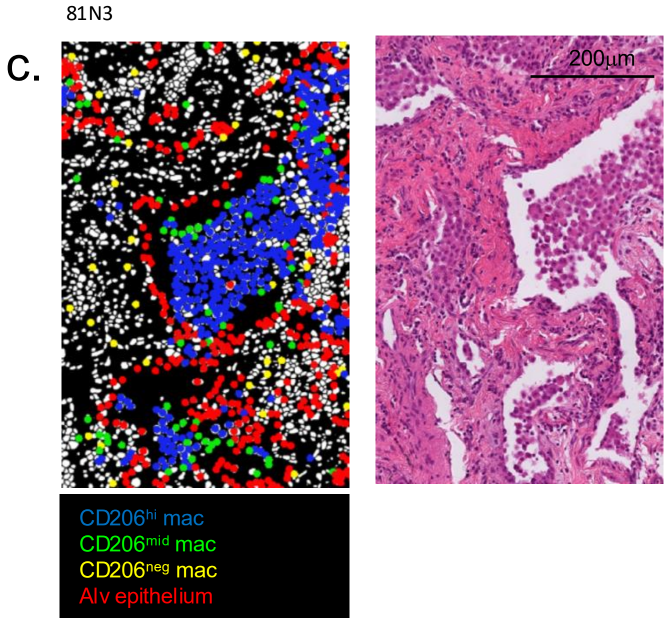

Supplementary Fig. 5. CD206^{hi} macrophages. a -c – cell centroid maps showing presence of CD206^{hi} (blue) and CD206^{mid} (green) macrophages within lumen of alveoli and CD206^{neg} macrophages (yellow) less frequent in interstitium. **b.** CD206^{hi} macrophages is distinctive on H&E sections, with basophilic cytoplasm and clustering, often found with CD206^{mid} macrophages, and they demonstrate a clearly higher level of CD206 expression than interstitial CD206^{neg} macrophages (**d**). Alv – alveolar, mac - macrophages

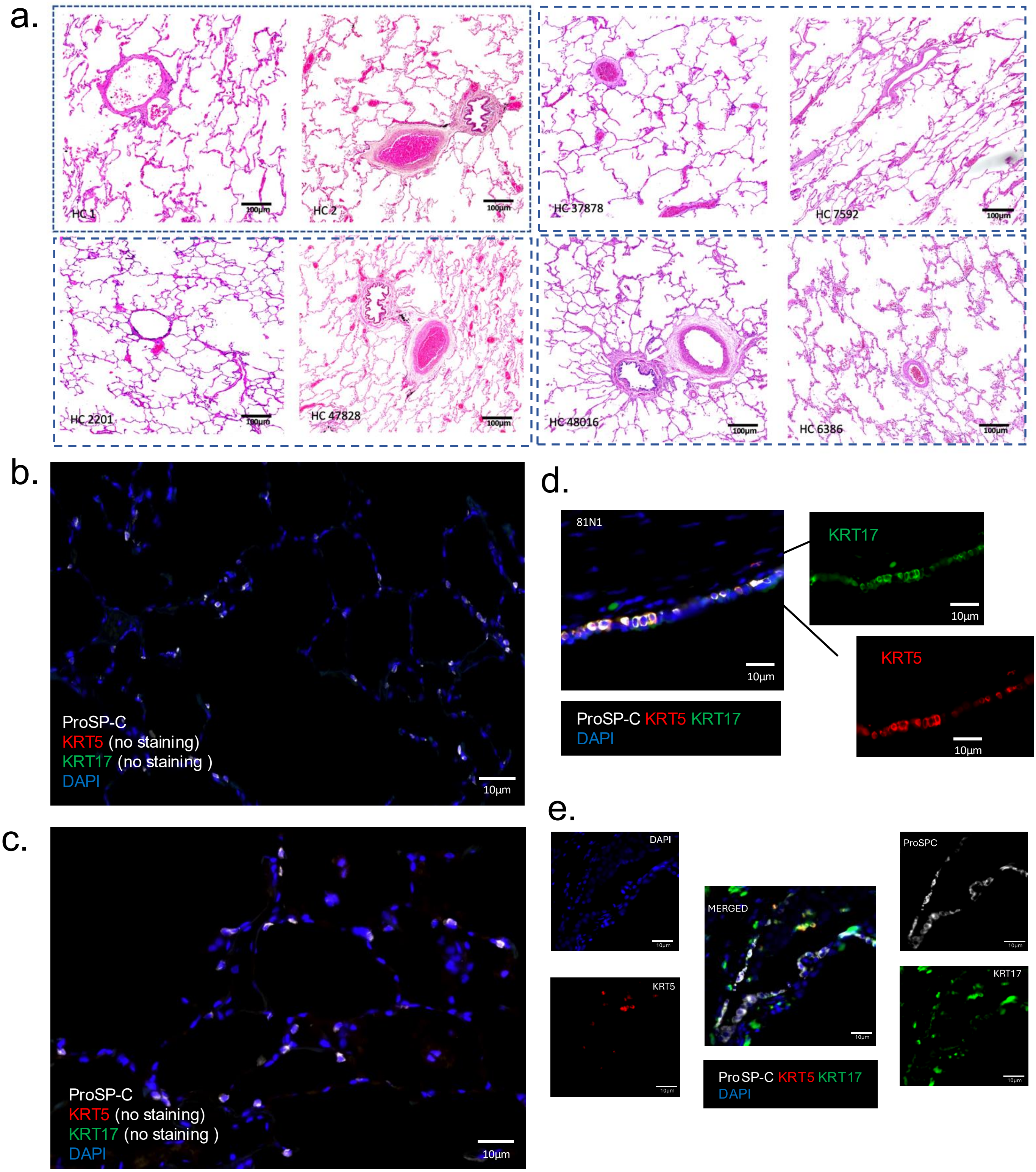

Supplementary Fig. 6. Healthy lung sections. a. H&E sections from 8 lung sections from healthy areas of lobectomised lungs in patients undergoing lung lobectomy for lung cancer (n=8 patients). Typical air spaces in alveolar units with thin epithelial lining is observed, with very little immune cell infiltrate in comparison to IPF lungs shown in Fig 1L-M where there is expanded interstitium, abnormal alveolar epithelial cells and dense cellular infiltrate. **b-c.** Immunofluorescence (IF) staining of healthy lung sections showing low frequency of ProSP-C^{+} cells (ATII cells) in alveolar epithelium and no KRT5 or KRT17 staining as expected for healthy alveolar epithelium. KRT17 antibody here is specific for KRT17 as opposed to KRT17/7 on IMC. Note also spaced out AT II cells compared to Suppl Fig 2B. **d.** ProSP-C, KRT5 and 17 staining in two IPF lung sections (**e-f**). All lung sections shown (**b-e**) were stained at the same time.

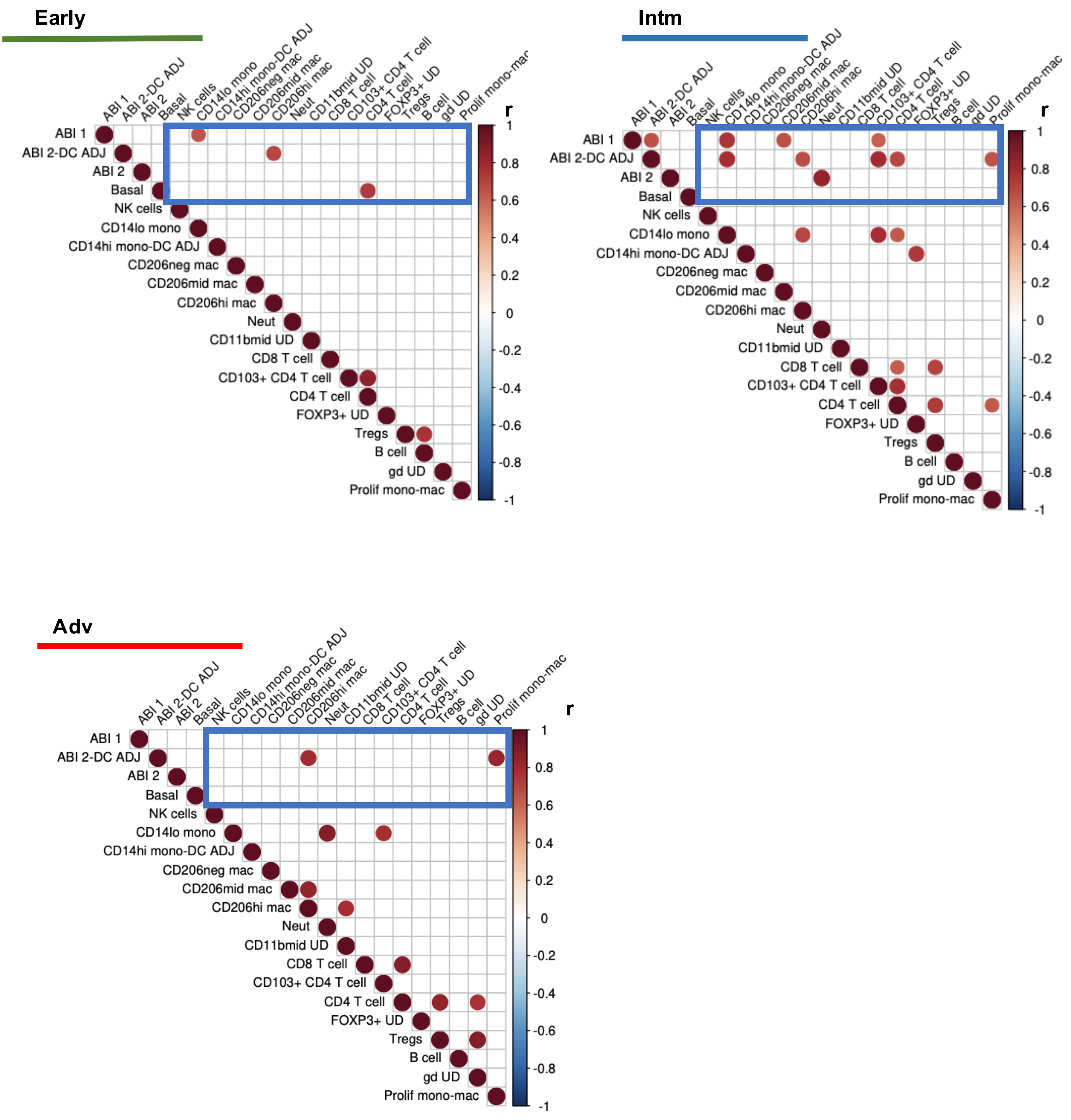

Supplementary Fig. 7 Correlation in abundance of cell types. Correlation in abundance between all cell types in across different disease stages, showing highest number of correlations between alveolar epithelial cell types and immune cell types (in blue box). Pearson correlation with Benjamini-Hochberg correction was used to analyse correlation; only Adj p< 0.05 is shown (as bubbles). r values are shown on right on colour gradient. Source data are available in Source Date File.

Supplementary Fig. 8

a.

Cross PCF $g_{C_1C_2}(r)$ for cells of types C_1 and C_2

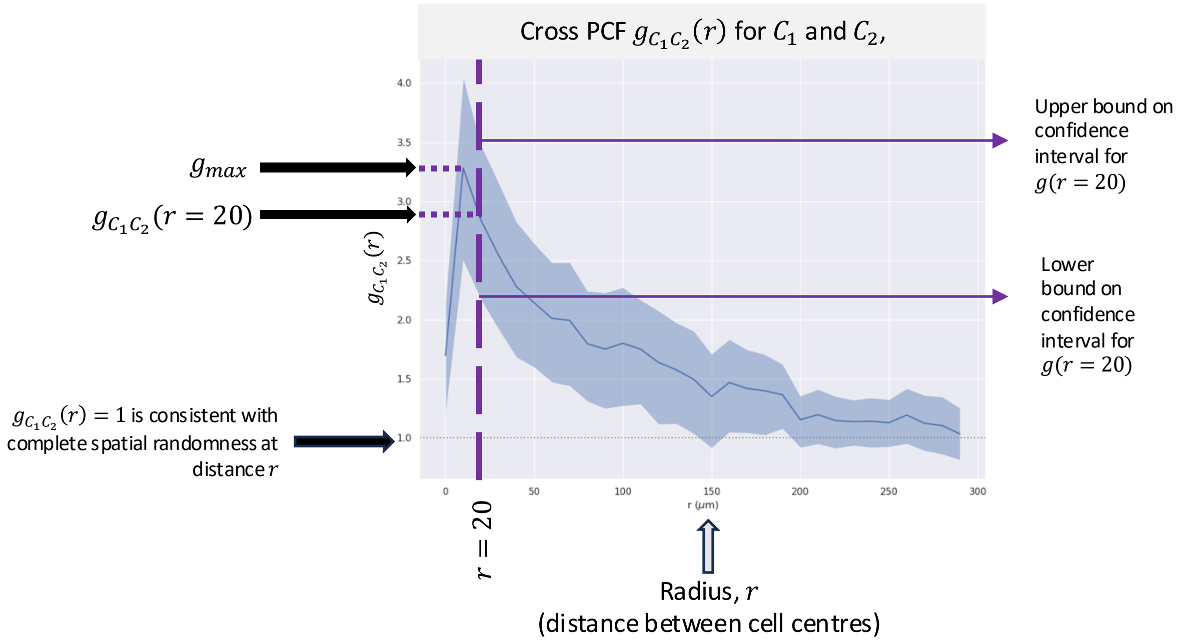

$$g_{C_1C_2}(r) = \frac{1}{N_{C_1}} \sum_{i=1}^{N} \mathbb{I}(C_1, c_i) \left(\sum_{j=1}^{N} \mathbb{I}(C_2, c_j) \frac{I_{[0,dr)}\left(|\boldsymbol{x}_i - \boldsymbol{x}_j| - \boldsymbol{r}\right)}{A_r(\boldsymbol{x}_i)} \Big/ \frac{N_{C_2}}{A} \right)$$

where:

- $N_{C_i} = \sum_{j=1}^{N} \mathbb{I}(C_i, c_j)$
- $\mathbb{I}(C, c_i) = 1$ if $c_i = C$, or 0 otherwise
- $I_{[a,b)}(r) = 1$ if $r \in [a, b)$, or 0 otherwise (for $a < b$ and $a, b \in \mathbb{R}$)

b.

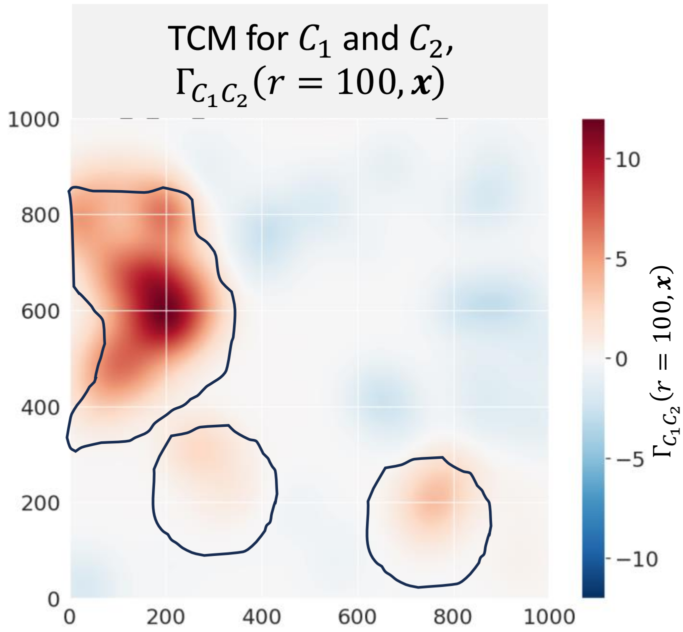

Highlighted regions depict co–location of C_1 and C_2 cells

$$\Gamma_{C_1C_2}(r, \boldsymbol{x}) = \sum_{i=1}^{N_{C_1}} \frac{\mu_{C_1C_2}}{2\pi\sigma^2} e^{-\frac{1}{2}\left(\frac{|x - x_i|}{\sigma}\right)^2}$$

where:

- $\boldsymbol{x}_i$ is the coordinate of cell i
- $\mu_{C_1C_2}(r, \boldsymbol{x}_i)$ is a normalised representation of the contribution of cell i to the cross-PCF at radii in the range from 0 to r
- σ is a parameter relating to the smoothness of the TCM

Supplementary Fig. 8 . Cross-PCF. a. Representative schematic of the cross-PCF $g_{C_1C_2}(r)$ for two cell types denoted C_1 and C_2, with the x-axis representing a length scale r that extends from the centroid of an index cell (C_1). For simplicity of notation, in the main text, we often write $g(r)$, the cell label subscripts being understood from the context. The y-axis shows the cross−PCF $g_{C_1C_2}(r)$, a spatial statistic that quantifies the degree of co-location between two cell types compared to that expected under an assumption of complete spatial randomness (CSR). This statistic measures how many times more frequently cells of type C_2 are observed in an annulus of inner radius r (and outer radius $r + 10\mu m$) centred around index cells of type C_1 than would be expected under CSR. We highlight $g_{C_1C_2}(r = 20)$, which is the relative over- (or under-) abundance of $C_1 - C_2$ cell pairs separated by distances in the range 20 to 30 microns. Also shown are $g_{max} = g(r_{max})$, the maximum value of $g_{C_1C_2}(r)$ over all r, and the radius $r = r_{max}$ at which it is attained. The 95% confidence intervals around$g_{C_1C_2}(r)$ are shaded in blue, and are derived via spatially blocked bootstrapping following [19] **b.** The topographical correlation map (TCM), $\Gamma_{C_1C_2}(r, x)$, superimposes a topographical representation onto the ROI to visualise the local strength of positive or negative spatial association between cells of types C_1 and C_2 across the ROI[20]. Here, and throughout this paper, we consider only $r = 100\mu m$ and use the shorthand $\Gamma_{C_1C_2} = \Gamma_{C_1C_2}(r = 50, x)$ for notational simplicity, where $x = (x, y)$ is a spatial location within the ROI. A positive value of $\Gamma_{C_1C_2}$(red shaded regions) indicates colocation of cells of type C_2 around cells of type C_1, while a negative value (blue shaded areas) indicates dispersal (presence of cells of type C_1 with relative sparsity of cells of type C_2 within $100\mu m$)[20].

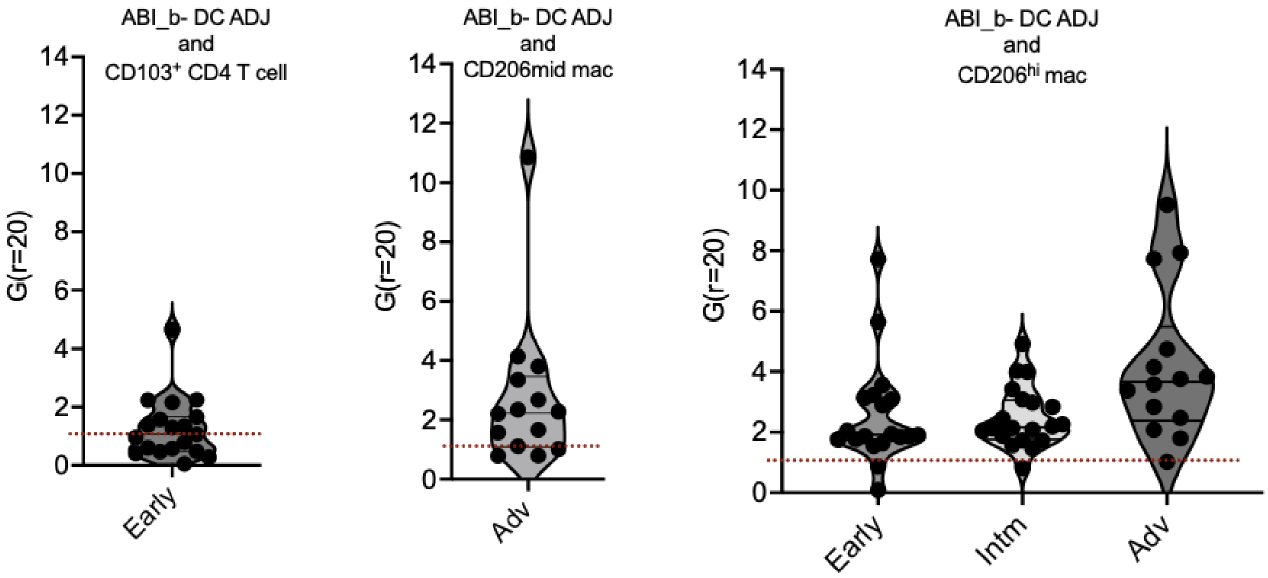

Supplementary Fig. 9 Distribution of g(r=20) values. Violin plots showing the distribution of g(r=20) values for cell type pairs identified as significantly co – located [g(r=20)>1] in the given disease states – see also figure 3B in main manuscript**.** Source data are available in Source Date File.

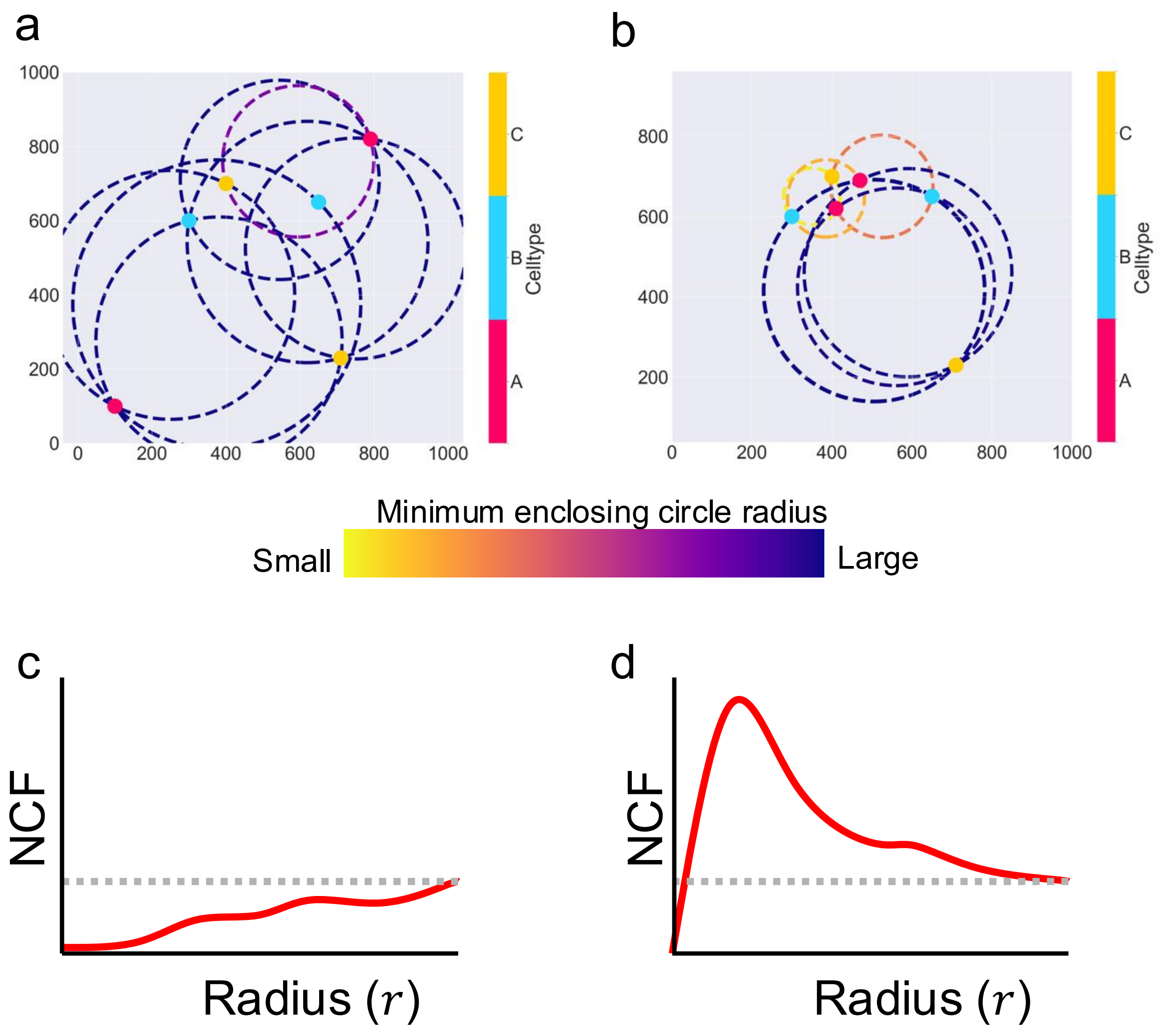

Supplementary Fig. 10 a. Neighborhood Correlation Function. a. Schematic showing three types of points (red, yellow, blue) which are not located in the same region of the domain, and thus have large minimal enclosing circles (MEC).**b.** The red points (cell type A) are in close proximity to the blue and yellow points (cell types B and C), leading to minimal enclosing circles with small radii. **c.** Schematic of an NCF that could describe 3-wise exclusion between cells of types A, B and C of the kind displayed in panel A - there are fewer MECs with small radii than would be expected for points distributed under complete spatial randomness. **d.** Schematic of an NCF that could correspond to 3-wise correlation of cells, with a large peak indicating more points within small neighbourhoods than would be expected under CSR

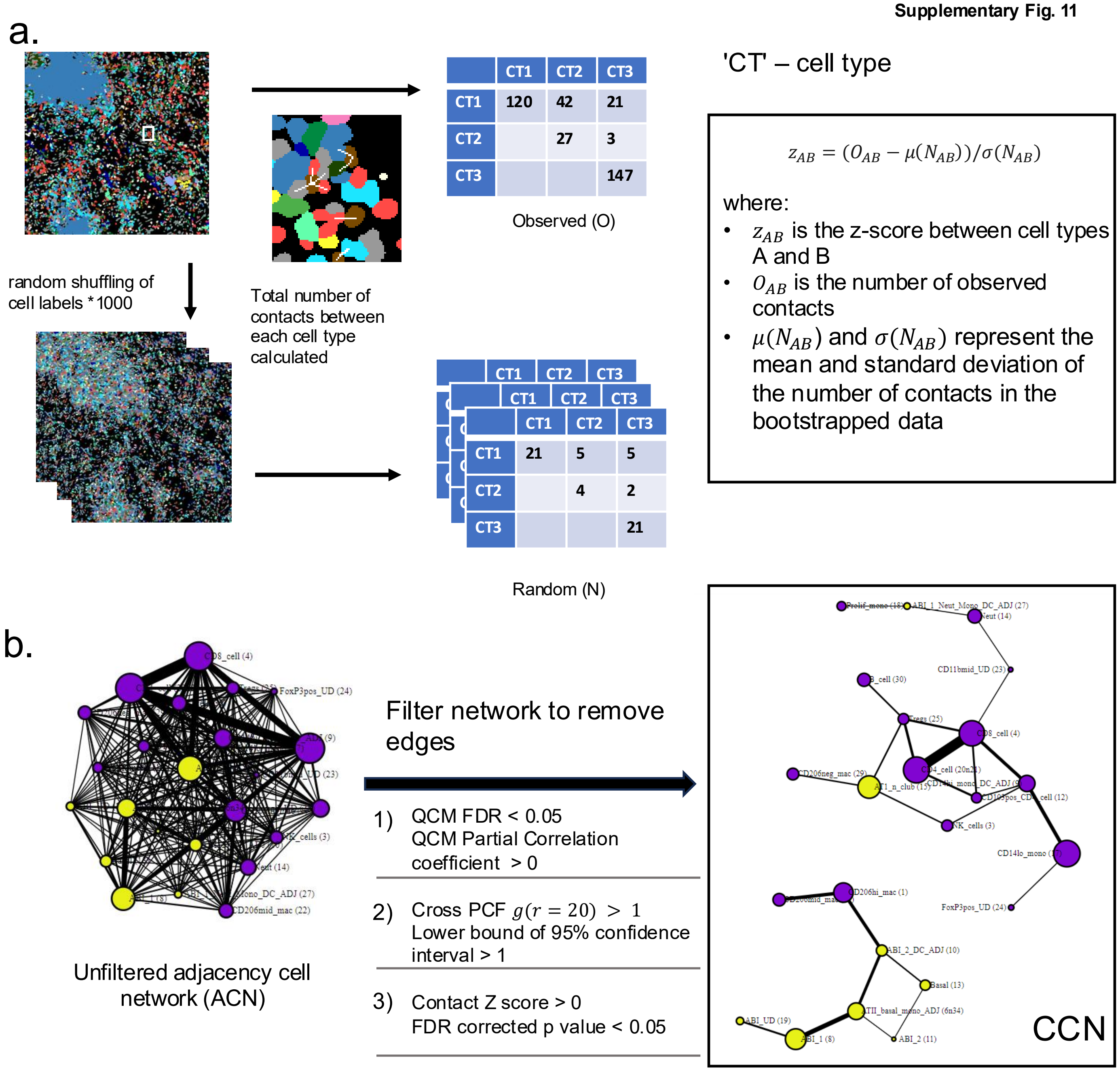

Supplementary Fig. 11 A. Workflow for derivation of adjacency contact cell networks (CCNs). a. Workflow for derivation of contact cell networks (CCNs). The segmentation mask generated by DeepCell is used to generate a spatially embedded network in which nodes are cell centres labelled according to cell type, and edges identify pixel-pixel contact between adjacent cells (adjacency cell network, ACN). We construct a matrix O of observed numbers of contacts between each pair of cell types. 1000 random networks are generated by permuting the labels of the ACN randomly, and a matrix N of numbers of contacts constructed for each random network. A contact Z score denoting deviation of the observed total contacts between cell types of interest is calculated from the mean and standard deviation of the 1000 matrices N, using the stated formula. FDR corrected p values are computed from these z scores. **b.** An unfiltered contact cell network is represented graphically, with nodes denoting different cell types and edges weighted by the numbers of contacts between them. The CCN is determined by filtering edges from this network using the following procedure:

1) (QCM FDR) < 0.05 and (QCM partial correlation coefficient) > 0
2) Cross PCF $g(r = 20) > 1$ and the lower bound of the 95% confidence interval of cross PCF $g(r = 20) > 1$ to select for statistically significant co–locating cell pairs.
3) (Contact Z score) > 0 and (FDR corrected p-value) < 0.05 to select cell pairs with significantly higher (more) contacts compared to random

Patient ID	Diagnosis	Age	Lung function tests		
			**FEV1(% of predicted)**	**FVC (% of predicted)**	**TLCO (% of predicted)**
RB81	IPF	60-70	2.29 (64%)	3.02 (66%)	2.87(28%)
RB82	IPF	50-60	2.10 (82%)	2.44(81%)	1.70(21%)
RB86	IPF	50-60	2.83(91%)	3.42(89%)	2.12(24%)
RB98	IPF	60-70	2.10(64%)	2.32(56%)	1.76(19%)
RB104	IPF	50-60	1.33(46%)	1.51(45%)	2.21(25%)
RB108	IPF	60-70	2.61(59%)	3.30(54%)	1.46(19%)
RB113	IPF	50-60	2.10(56%)	2.55(55%)	2.45(23%)
RB051	HC	20-30	N/A	N/A	N/A
RB052	HC	40-50	N/A	N/A	N/A

Supplementary Table 1. Demographic of patients at the point of sample harvesting. 71% of IPF patients are male and both HC are male. Aggregated sex data provided to prevent identification of patients.
N/A – Not available

Disease Stage and ROI identifier	cell_number	sample_id	Overall histopathology picture *	Fibroblastic foci (FF) (0=none; 1=present)	Number of FF	Bronchiolisation (0=none; 1=present)	
**Advanced**							
25	5695	IPF_SAMPLE_081F_ROI_1	C	**0**	0	0	**n=14 ROI**
33	5493	IPF_SAMPLE_081F_ROI_2	A/B	**0**	0	0	**10 C, 2 B/C, 1 A/B, 1 B**
12	4915	IPF_SAMPLE_086F_ROI_1	C	**0**	0	0	**71%C, 14% B/C**
45	5011	IPF_SAMPLE_086F_ROI_2	C	**0**	0	0	**14% of ROIs have FF**
44	10198	IPF_SAMPLE_098F_ROI_1	B/C	**1**	1	0	**total cells = 89826**
26	11193	IPF_SAMPLE_104F_ROI_2	C	**0**	0	0	
18	4477	IPF_SAMPLE_108F_ROI_1	C	**0**	0	1	
48	6344	IPF_SAMPLE_113F_ROI_1	C	**1**	2	0	
55	6663	IPF_SAMPLE_113F_ROI_2	C	**0**	0	0	
11	5252	IPF_SAMPLE_113F_ROI_3	C	**0**	0	0	
0	5390	IPF_SAMPLE_113F_ROI_4	C	**0**	0	0	
59	7416	IPF_SAMPLE_113F_ROI_5	C	**0**	0	0	
42	5080	IPF_SAMPLE_82F_ROI_2	B	**0**	0	0	
27	6699	IPF_SAMPLE_82F_ROI_3	B/C	**0**	0	1	
**Intm**							
35	6179	IPF_SAMPLE_081I_ROI_1	B	**1**	2	0	**n=20 ROI**
3	4989	IPF_SAMPLE_081I_ROI_2	B	**1**	1	0	**2 C, 4 B/C, 9 B, 2 A/B, 3A**
46	6388	IPF_SAMPLE_081I_ROI_3	B	**1**	2	0	**45%B, 20% B/C**
56	4977	IPF_SAMPLE_081I_ROI_4	B	**0**	0	0	**55% has FF**
57	4720	IPF_SAMPLE_086I_ROI_1	A	**1**	3	0	**total cells = 140278**
20	4724	IPF_SAMPLE_086I_ROI_2	A	**1**	3	0	
50	4991	IPF_SAMPLE_086I_ROI_3	A	**0**	0	0	
43	9858	IPF_SAMPLE_098I_ROI_1	B	**1**	3	0	
60	9783	IPF_SAMPLE_098I_ROI_2	B	**1**	2	0	
14	10026	IPF_SAMPLE_098I_ROI_3	A/B	**0**	0	0	

34	9775	IPF_SAMPLE_098I_ROI_4	B/C	**1**	1	0	
32	9841	IPF_SAMPLE_098I_ROI_5	B/C	**1**	3	0	
39	9852	IPF_SAMPLE_104I_ROI_1	B/C	**0**	0	0	
36	7734	IPF_SAMPLE_104I_ROI_2	B	**1**	1	1	
16	6127	IPF_SAMPLE_108I_ROI_1	C	**0**	0	0	
23	6410	IPF_SAMPLE_108I_ROI_2	B/C	**0**	0	0	
61	4186	IPF_SAMPLE_113I_ROI_1	A/B	**1**	1	0	
24	7717	IPF_SAMPLE_113I_ROI_2	B	**0**	0	0	
29	6157	IPF_SAMPLE_113I_ROI_3	B	**0**	0	0	
10	5844	IPF_SAMPLE_113I_ROI_5	B/C	**0**	0	0	
**Early**							
22	4289	IPF_SAMPLE_081N_ROI_1	C	**0**	0	0	**n=19 ROI**
53	5104	IPF_SAMPLE_081N_ROI_2	C	**0**	0	0	**5 C, 5 B/C, 1B, 3 A/B, 5A**
38	6305	IPF_SAMPLE_081N_ROI_3	B/C	**0**	0	0	**26% A, 26%B/C, 26% C**
9	5715	IPF_SAMPLE_081N_ROI_4	B/C	**1**	1	0	**47% has FF**
6	7602	IPF_SAMPLE_098N_ROI_1	C	**0**	0	0	**total cells = 112406**
2	9575	IPF_SAMPLE_098N_ROI_2	C	**0**	0	0	
52	7256	IPF_SAMPLE_098N_ROI_3	B	**1**	2	0	
8	7033	IPF_SAMPLE_108N_ROI_1	M/L	**1**	1	0	
47	5603	IPF_SAMPLE_108N_ROI_2	C	**0**	0	0	
30	6599	IPF_SAMPLE_113N_ROI_1	A	**0**	0	0	
40	7194	IPF_SAMPLE_113N_ROI_2	A	**0**	0	0	
17	6307	IPF_SAMPLE_113N_ROI_3	A/B	**1**	3	0	
7	3938	IPF_SAMPLE_82N_ROI_1	A/B	**1**	2	0	
41	4410	IPF_SAMPLE_82N_ROI_2	B/C	**1**	2	0	
49	3726	IPF_SAMPLE_82N_ROI_3	B/C	**0**	0	0	
13	4422	IPF_SAMPLE_82N_ROI_4	A/B	**1**	2	0	
19	5315	IPF_SAMPLE_86N_ROI_1	A	**0**	0	0	
51	7120	IPF_SAMPLE_86N_ROI_2	A	**1**	3	0	
54	4893	IPF_SAMPLE_86N_ROI_4	A	**1**	1	0	

Supplementary Table 2. Histopathology analysis of lung sections.

***Overall histopathology picture**

a. Collagen less prominent, more fibroblastic foci (FF), more inflammatory infiltrate, less expanded interstitium

b. More collagen, less FF, inflammatory infiltrate, more expanded interstitium

c. Most collagen, more prominent smooth muscle hyperplasia, v little airspace left, little or no FF, occasional evidence of bronchiolisation

Marker	Metal
CD45	141Pr
CD68	142Nd
CD8a	143Nd
Ki67	144Nd
alpha-Smooth Muscle Actin	145Nd
FoxP3	147Sm
CD11c	149Sm
CD103	152Sm
CD56	153Eu
Helios	154Sm
CD69	155Gd
Epcam	156Gd
CD20	158Gd
CD206	159Tb
CD127	160Gd
KRT17	161Dy
CD1c	162Dy
CD15	163Dy
KRT14	164Dy
TCRγ	165Ho
Amphiregulin	166Er
CD11B	167Er
BDCA2	168Er
KRT5	169Tm
CD3	170Er
proSP -C	171Yb
CD31	172Yb
CD161	173Yb
CD4	174Yb
HLA-DR	175Lu
CD14	176Yb
DNA - 1	191Ir
DNA - 3	193Ir

Supplementary table 3 – 33 plex panel with their metal tags for Imaging Mass Cytometry.

A.

% of all cells	Cluster	Name	Description and expanded notes
1.60%	3	**NK cells**	CD56^{+} population.
10.30%	17	**CD14^{lo} mono**	All cells are CD14^{lo} and HLADR ^{mid} and CD68 $^{neg.}$ Likely patrolling monocytes found on or near endothelium of blood vessels(*1*). Spatial analysis in our work shows co-location with vasculature.
3.10%	9	**CD14^{hi} mono-DC ADJ**	CD14^{hi} – the only CD14^{hi} cluster - classical monocytes (CD14^{hi}CD16^{neg} monocytes)(*2*). Likely found with DC adjacent to it, due to CD11c^{hi}, CD1c^{hi} and HLADR^{hi} expression. Can also be transitional macrophages (transitioning between monocytes to macrophage in differentiation pathway)(*2*). See also Suppl Figure 4B for staining.
1.30%	29	**CD206^{neg} mac**	CD68^{lo} CD206^{neg}CD14 ^{neg} HLADR ^{neg}; likely interstitial macrophage due to location of cells. See Suppl Fig 5A-C for location in cell centroid maps.
1.60%	22	**CD206^{mid} mac**	CD68^{mid}CD206^{mid}CD14^{neg}HLADR^{mid}; alveolar macrophages. Always found with CD206^{hi} macrophages in alveolar lumen; note CD68 expression lower suggesting earlier in differentiation trajectory from monocyte to macrophages but they are CD14^{neg}. Spatial co-location with CD206^{hi} mac supports this possibility of less mature alveolar macrophage (Fig 5D-F). See Suppl Fig 5A-C for location in cell centroid maps.
5.90%	1	**CD206^{hi} mac**	CD68^{hi} CD206^{hi} CD14^{mid} HLADR^{hi}. Alveolar macrophages. Most mature macrophage with high HLA DR and CD68. See Suppl Fig 5A-C for location in cell centroid maps.
3.70%	14	**Neut**	Neutrophils. Only CD15 expressing cells found
0.30%	23	**CD11b^{mid} UD**	Clear CD11b expression but no other defining features. Identity unknown.
6.90%	4	**CD8 T cell**	CD69 ^{neg} CD11b ^{neg} CD103 ^{neg} CD8 T cells
1.10%	12	**CD103^{+} CD4 T cell**	CD68 ^{neg} CD103^{+} CD4 T cells. Likely resident CD4 T cells; usually found in alveolar lining.
7.10%	20	**CD4 T cell**	CD69 ^{neg} CD4 T cells
2.20%	21	**CD4 T cell**	CD69 neg CD4 T cells
0.30%	24	**FOXP3^{+} UD**	FOXP3 $^{mid\text{-}hi}$ CD3 ^{neg} CD4 l^{o} cells HELIOS^{mid} AREG$^{lo.}$ Identity unclear.
1.10%	25	**Tregs**	FOXP3 $^{lo\text{-}mid}$ CD3 $^{mid\text{-}hi}$ CD4 $^{mid\text{-}hi}$ HELIOS ^{mid} AREG ^{lo}
4.50%	30	**B cell**	CD20 $^{mid\text{-}hi}$
3%	26	**γδ UD**	CD45 ^{neg} and EPCAM ^{neg}, could be non-specific staining
1.20%	18	**Prolif mono-mac**	Proliferating monocyte-macrophage. Almost identical to cluster 17 (CD14^{lo} mono) - all cells are CD14^{lo} , HLADR ^{mid} and Ki67 $^{mid\text{-}hi}$. Only cluster to show this high level of Ki67 expression
5.50%	15	**15 UD**	CD45^{lo}, EpCAM^{lo}, ProSPC^{neg}, KRT5^{neg}, KRT17/7^{neg} - spatial check on cell centroid map and IMC images suggest these are immune cells found scattered in interstitium. Some cells found close to ABIs. Likely immune cells with some cells adjacent to ABIs. Included in immune cell group. Unlikely to contain ATI as 15 UD cluster is KRT17/7^{neg} while Haberman's dataset (Suppl Fig 3B clearly shows ATI expressing high levels of KRT 7)

10.10%	5	**Vasculature**	$CD31^{mid}$, $\alpha SMA^{neg\text{-}lo\text{-}mid}$. A mix of arterioles, venules and lymphatics
0.20%	28	**Endothelium**	$CD31^{lo}$, mainly capillaries in alveolar bed
5.40%	2	**SM**	Smooth muscle. αSMA^{hi} $CD45^{neg}$ and $EPCAM^{neg}$ - could be fibroblasts, smooth muscle cells as part of SM hyperplasia in IPF or smooth muscle around arteries or bronchioles
4.90%	6	**ATII**	AT II cells (high proSP-C expressing cells); mainly $KRT5^{neg}$, KRT17/7 positive on IMC (likely due to KRT7 positivity). Immunofluorescence staining shows negative KRT17 staining (Suppl Fig 3E) Final Cluster 6 included both cluster 6 and 34
5.30%	8	**ABI_a**	Early ABI in trajectory between $KRT5^{-}KRT17/7^{+}$ ABI and basal cells
1.90%	10	**ABI b-DC ADJ**	Mid ABI in trajectory between $KRT5^{-}KRT17/7^{+}$ ABI and basal cells, adjacent to DCs which can be in lumen or interstitium; note it is the only ABI with low CD45 expression in keeping with presence of immune cell (DC) (Suppl Fig 2A; Fig 2F). Does not discriminate between DC and CD11c-expressing macrophages but called DC due to higher expression of CD1c and lower expression of CD68 and CD206 (see Suppl Table 4B below).
0.40%	11	**ABI_b**	Mid ABI in trajectory between $KRT5^{-}KRT17/7^{+}$ ABI and basal cells. Note very small numbers of cells.
1.70%	13	**Basal**	$KRT5^{hi}$ $proSP\text{-}C^{neg}$ cells. Likely end point of ABI differentiation trajectory. Staining shown in Suppl Fig 3F
1.30%	19	**19 UD**	$EpCAM^{neg}$, $CD45^{neg}$, $KRT5^{lo}$, $KRT17/7^{neg/lo}$ - identity unclear
0.40%	27	**27 UD**	$EpCAM^{neg\text{-}lo}$,$ProSP\text{-}C^{lo}$, $KRT5^{neg\text{-}lo}$, $KRT17/7^{mid}$, $CD14^{lo}$, $BDCA2^{mid}$, $CD11b^{mid}$,$CD15^{mid}$, $CD14^{lo}$, $CD1c^{lo\text{-}mid}$. Likely mix of immune cells (neutrophils, monocytes, DC) with an ABI , ATI or AT II. Very small numbers of cells. Spatial co-location shows spatial association with Neutrophils in all disease stages (Fig 5D-F). Included in epithelial cell group
0.04%	34	**ATII**	AT II cells (high proSP-C expressing cells) – merge with Cluster 6 and overall called Cluster 6.

1. C. Auffray et al., Monitoring of Blood Vessels and Tissues by a Population of Monocytes with Patrolling Behavior. Science 317, 666-670 (2007).
2. E. Fraser et al., Multi-Modal Characterization of Monocytes in Idiopathic Pulmonary Fibrosis Reveals a Primed Type I Interferon Immune Phenotype. Front Immunol 12, 623430 (2021).

B.

Myeloid cell clusters and selected epithelial cells and their relative expression of relevant markers								
**Name**	**Cluster number**	**CD11c**	**CD14**	**CD1c**	**HLADR**	**CD68**	**CD206**	**BDCA2**
**CD206^{hi} mac**	1	+	++	+++	+++	+++	+++	+
**CD14^{hi} mono-DC ADJ**	9	++	+++	+++	+++	+/-	-	+
**CD14^{lo} mono**	17	-	++	+	++	-	-	-
**Prolif mono-mac**	18	-	++	+	++	-	-	+
**CD206^{mid} mac**	22	+	-	+	++	++	++	-
**CD206^{neg} mac**	29	-	-	-	-	+/-	-	-
**ABI b-DC ADJ**	10	+	++	+++	+++	+/-	+/-	+
**Basal**	13	-	++	+++	+/-	-	-	+
**27 UD**	27	-	++	+	+/++	-	-	+
**ATII**	6	-	++	+	+++	-	-	+

Supplementary Table 4. Annotation of clusters, and expanded notes on annotation.

Target	Ab Clone	Vendor	Cat no.	Antibody Registry	Lot.	Metal
CD45	D9M8I	Cell Signal Technology	13917BF	AB_2750898	11	141
CD68	KP1	Biolegend	916104	AB_2616797	B283618	142
CD8a	C8/144B	Biolegend	372902	AB_2650657	B298974	143
Ki67	Polyclonal	Novus	NB500-170	AB_10001977	H-2	144
alpha-Smooth Muscle Actin	1A4/asm-1	Novus	NBP2-33006	AB_3094464	IBR082005B	145
FoxP3	236A/E7	Abcam	ab20034	AB_445284	GR3443434-2	147
CD11c	2F1C10	Protein Tech	60258-1-Ig	AB_2881379	10002357	149
CD103	EPR22590-27	Abcam	ab254201	AB_2891141	GR3288218	152
CD56	E7X9M	Cell Signal Technology	99746	AB_2868490	2	153
Helios	E4L5U	Cell Signal Technology	89270	AB_3094463	2	154
CD69	15B5G2	Cell Signal Technology	NBP2-25236	AB_3094462	0320453162-03	155
Epcam	Polyclonal	Abcam	ab71916	AB_1603782	CR3357742-1	156
CD20	4A7G3	Protein Tech	60271-1-Ig	AB_2881391	1003534	158
CD206	2A6A10	Protein Tech	60143-1-Ig	AB_2144924	10004170	159
CD127	EPR2955(2)	Abcam	ab240225	AB_3094461	GR3375511-1	160
KRT7/17	C-46	Biolegend	628702	AB_439777	B254700	161
CD1c	2A7C11	Novus	NBP2-61726	AB_3094460	170831	162
CD15	SSEA-1	Biolegend	323002	AB_756008	B254011	163
KRT14	2G1E2	Protein Tech	60320-1-Ig	AB_2881431	1332030	164
TCRδ	H-41	Santa Cruz Biotechnology	91921	AB_1130061	sc-100289	165
Amphiregulin	1A1G9	Protein Tech	66433-1-IG	AB_2881803	10004459	166
CD11B	1C7C2	Protein Tech	66519-1-Ig	AB_2881882	10003969	167
BDCA2	10E6.1	Millipore	MABF94	AB_2889343	3698182	168
KRT5	2C2	Thermofisher	MA5-17057	AB_2538529	VJ3094812	169
CD3	PC Sera	Fluidigm	3170019-D	AB_2811048	2101806-28	170
ProSPC	Polyclonal	Novus	NPB1-60117	AB_11029499	KL63276-170622	171
CD31	EPR3094	Abcam	ab207090	AB_2889382	G-R3229164-12	172
CD161	OTI1D8	Abcam	ab273666	AB_3094458	GR33527516	173
CD4	EPR6855	Abcam	Ab181724	AB_2864377	GR3285644-12	174
HLA-DR	LN3	Biolegend	327002	AB_893582	B262251	175
CD14	EPR3653	Abcam	EPR3653	AB_2889158	GR33451137	176

Target	Ab Clone	Vendor	Cat no.	Antibody Registry	Conjugate	Working Concentration
Cytokeratin 17	EP1623	Abcam	AB185032	AB_2889195	Alexa Fluor® 488	1 in 200
CD15/SSEA1	MC480	Cell Signaling	#4744	AB_1264258	NA	1 in 200
Prosurfactant	Rabbit	Abcam	AB90716	AB_10674024	NA	1 in 400
Cytokeratin 5	EP1601Y	Abcam	AB193895	AB_2728796	Alexa Fluor®	1 in 200
MRC-1	CL0387	Atlas antibodies	AMAB90746	AB_2665652	NA	1 in 500
BDCA-2	10E6.1	Merck Millipore	MABF94	AB_2889343	NA	1 in 100
Fibronectin	F1	Abcam	AB198934	AB_3094466	Alexa Fluor®	1 in 200
Goat anti Rabbit	Polyclonal	Thermo Fischer	A21039	AB_10375716	Alexa Fluor®	1 in 300
Goat anti-mouse	Polyclonal	Thermo Fischer	A21144	AB_2535780	Alexa Fluor®	1 in 300
ProSPC	H-8	Santa Cruz	sc-518029	AB_2937075	NA	1 in 200
ki67	MIB-1	Agilent Dako	GA62661-2	AB_2687921	NA	1 in 100

Supplementary Table 5. Antibodies used in imaging mass cytometry and other validation sudies.

Name of software	Version	Source	Identifier
imctools	2.1.8	https://github.com/BodenmillerGroup/imctools	RRID:SCR_017132
Deepcell	0.12.4	https://vanvalen.github.io/about/	RRID:SCR_022197
Phenograph	1.5.2	https://github.com/JinmiaoChenLab/Rphenograph	RRID:SCR_016919
Harmony	0.1.1	https://github.com/slowkow/harmonypy	RRID:SCR_022206
Ruffus	2.6.3	http://www.ruffus.org.uk/	RRID:SCR_022196
QuPath	0.5.1	https://qupath.github.io/	https://doi.org/10.1038/s41598-017-17204-5
MCD	1.0.560.6	https://www.standardbio.com/products-services/software	RRID:SCR_023007
Catalyst R	1.32.0	http://bioconductor.org/packages/CATALYST/	RRID:SCR_017127
diffcyt R package (version 1.8.8)	1.8.8	https://www.bioconductor.org/packages/release/bioc/html/diffcyt.html	RRID:SCR_023006
Cellchat	1.6.1	https://github.com/jinworks/CellChat	RRID:SCR_021946
Seurat	4.3	https://satijalab.org/seurat/	RRID:SCR_007322

Supplementary Table 6. Software used in data processing and analysis

Supplementary Information

Extended quality control (QC) steps

Background. As a pre-requisite, the 31 antibodies for the IMC panel are chosen very carefully to answer the question posed (i.e. how immune cells behave temporally and spatially in regenerating alveolar niche in IPF). These antibodies are well established and are 'identifier' or unique phenotyping markers with high specificity for the cell type, and good binding properties. Selection of antibodies and optimisation of marker expression are done carefully before the experiment so that, at the point of QC for the expression data, very little data are filtered, unlike for scRNAseq. Biological knowledge is highly involved in analysis e.g. antibodies reflect prior biological knowledge in contrast to scRNAseq where transcriptome-wide markers allow greater scope for discovery.

Antibody validation, sample staining and acquisition of expression

Lanthanide metal conjugation of antibodies was validated by first checking the recovery of antibody post-conjugation. Then, we checked for successful metal conjugation by binding the antibody to iridium labelled antibody capture beads AbC™ Total Antibody Compensation Beads (Thermo Fisher, USA, CAT#A10513) and acquiring on a Helios system (cell suspension mode). Finally, we checked that the antibody had refolded and retained the ability to recognize antigen by using the post-conjugation antibody in either a two-layer immunofluorescence (IF) with a fluorescently-labelled secondary antibody recognizing the primary antibody species or directly by IMC using the Hyperion imaging module (tissue mode).

All antibodies were validated in healthy AND disease (IPF) lung and other organ sections (prostate, kidney), and healthy secondary lymphoid tissue (tonsils) and shown to work well in the Hyperion system at pH9 using the Heat Induced Epitope Retrieval (HIER) retrieval system[2]. The antibodies were further validated for use by single-plex immunofluorescence staining of lung sections from the same cohort. Positive tissue controls and no primary antibody controls were used in each instance (examples shown in Figure R1).

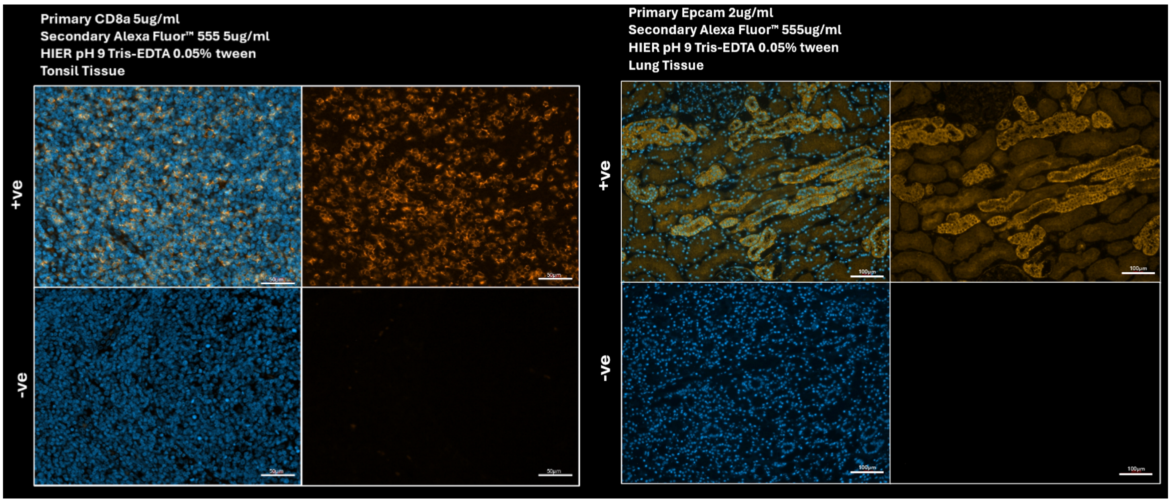

Figure R1. *Immunofluorescence staining of tonsil (left) and lung (right) tissue with exemplar antibodies before testing with metal tagged antibodies in IMC.*

An important step in QC is the systematic correction for any signal spill-over between excitation channels[3]. This is typically very small in mass cytometry and further minimised by careful optimisation and selection of isotopes and antibody concentrations. However, spill-over can be present and can complicate interpretation of data. For example, signal crosstalk can result in incorrect identification of cells as expressing an intermediate level of a marker. In our experiment, polystyrene capture beads were single stained with each antibody used in the experiment, analyzed simultaneously in the mass cytometer. The CATALYST R/Bioconductor package (https://bodenmillergroup.github.io/IMCDataAnalysis/spillover-correction.html)[4], was used to deconvolute the different bead populations, estimate spillover signal in all channels, and compensate the data.

Once these QC steps had been performed, IMC staining was performed in a minimal number of batches (in our case, three), in equally balanced groups [balanced for Early, Intm and Advanced lung sections, healthy control lung sections and on the day, a positive control sample (tonsils) and negative controls were used for each batch].

Acquisition [i.e. ablation of tissue by the tissue mass cytometer (Hyperion)] was done on one machine sequentially over 2 weeks and all checks for technical stability of the machine were performed between and again, just before each acquisition. Prior to each set of ablation, the Hyperion Tissue Imager was calibrated and rigorously quality controlled to achieve reproducible sensitivity based on the detection of one of the isotope (^{175}Lutetium). In brief, a stable plasma was allowed to develop prior to ablation of a multi-element-coated "tuning slide" (available from Standard Biotools). During this ablation, performance was standardized to an acceptable range by optimizing system parameters using the manufacturer's "auto-tune" application or by manual optimization of XY settings whilst monitoring ^{175}Lutetium dual counts. Small regions of tonsil tissue were first targeted to ensure complete ablation of tissue during the laser shot with ablation energies adjusted to achieve this where required.

Data processing and filtering:

QC steps for expression data

a. Expression signals from all 31 antibodies and 2 DNA markers (in the form of a TIFF file) are extracted from the imaging files (MCD) for each excitation channel.
b. The data are first examined for spillover of signal between excitation channels – these should be less than 5%. We found no spillover due to prior optimization of antibodies and metal tag allocation to appropriate channels as described above.
c. Expression of each antibody is visually examined in <u>all</u> ROIs using MCD viewer of the images, compared to H&E sections; and a 'first pass' list of antibody filter is performed as follows:
    i. Antibodies which show diffuse staining in all cells without discrimination for appropriate cells or which showed very low levels of expression in all samples including positive control on day of staining, are excluded from further use. The following antibodies were excluded from analysis - BDCA2, KRT14 (see Figure R2)
    ii. Antibodies showing no staining at all. None were excluded

d. First pass segmentation of cells on images is performed using a computational analytical algorithm (Deep Cell). The average intensity of each marker for each cell is obtained and submitted for unbiased dimensionality reduction and clustering process with R – Phenograph. All samples are integrated, harmonized and batch corrected (using Harmony[5]) to minimize individual sample and batch effects (as in scRNAseq analysis), and an overall representation

of the marker expression per cluster across all samples (expression density histogram) is then generated (Suppl Fig 1i). Annotation as described in the paper (Suppl Fig 1a-g).

e. At this point, three specific QC steps are undertaken:
   i. A cell cluster that does not show any antibody expression is identified. This is usually because none of the 31 antibodies are found on the cells. These are excluded from further analysis. 7.9% of total cells from all samples fell into this category.
   ii. Cell clusters that comprise less than 0.1% of total cells were excluded. There were 5 such cell clusters.
   iii. A cluster is termed undefined (UD) if there is insufficient information to annotate its constituent cells and if they expressed markers which are biologically implausible e.g. defining markers in epithelial cells AND immune cells. 6 such clusters were identified, and comprised 10.8% of the total cells from all samples. These information can be found in Suppl Table 4.

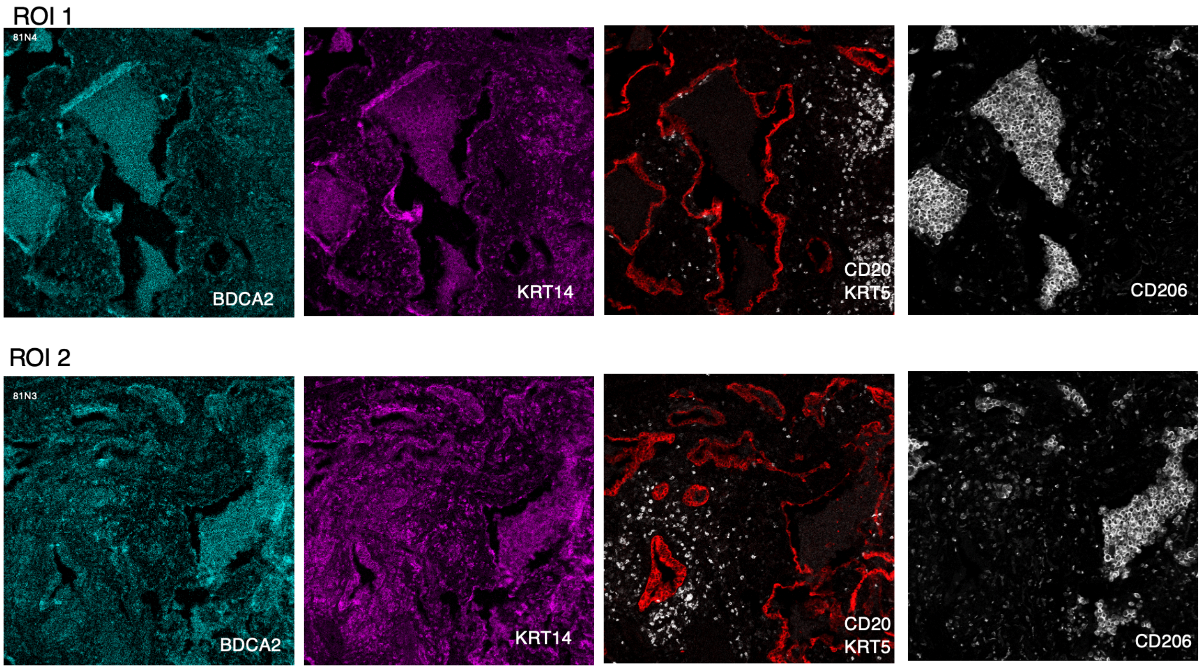

Figure R2. *MCD file expression images showing the expression of low quality staining (BDCA2 and KRT14) and good quality staining (KRT5, CD20 and CD206)in IMC, in two representative IPF regions of interest (upper and lower panels)*

Expanded information on tissue harvest, preparation and analysis of signal expression

Tissue factors. A very rigorous and well-established protocol designed to minimize variation in tissue harvesting and processing is in place. Only one transplant physician and one lung pathologist are involved in tissue harvesting (Newcastle Lung Transplant Centre), there is a well established harvest, store and lung sectioning pipeline (Newcastle histopathology research pipeline), and a direct pathway from patient to bench, using standardised research SOP. Acquisition was done on one Hyperion machine sequentially over 2 weeks, in three balanced batches and all checks for technical stability of the machine were performed before (and during) each acquisition.

Each H&E stained, next-in-sequence lung section is reviewed by a senior pathologist (>30 years experience) and two senior pulmonologists (>20 years experience) to identify regions of interest (ROIs). Small ROIs were used (1x1mm), allowing us to choose areas that demonstrate excellent

sectioning without folds and with consistent staining, enabling us to avoid areas with mucus impaction and high numbers of red blood cells which can affect staining.

Optimal thickness of lung sections was pre-determined. Performing multiple rounds of staining using antibodies enabled us to select a tissue thickness that most likely corresponds to a single cell. For example, CD3 T cells are either CD4+CD8- (CD4 T cells) or CD4-CD8+ (CD8 T cells) in healthy tissue. An optimal lung thickness will capture these two cell types as distinct entities, while a section that is too thick may capture a false cell cluster of CD4+CD8+ cells if these two cell types are on top of each other (Figure R3).

These well established, tested and standardised pre-staining provisions and optimisation minimise the kind of problems that this reviewer has justifiably raised.

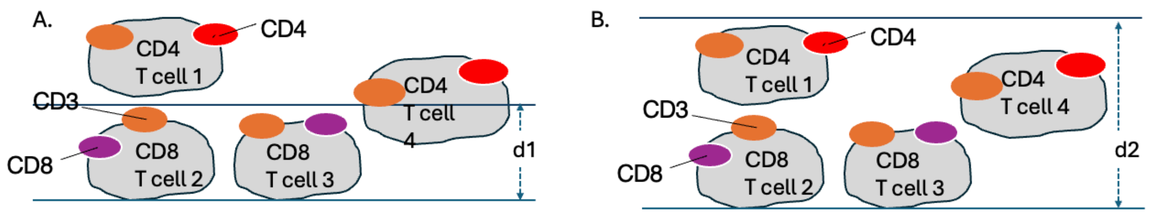

A. d1 – thickness of sectioned tissue
This is correct thickness as biologically, CD3 T cells should be either CD3+CD4+ (CD4 T cells) <u>or</u> CD3+CD8+ (CD8 T cells). There should be an approximate 1:1 ratio of CD4 and CD8 T cells in healthy lungs.

B. d2 – lung section is too thick and will show CD4+CD8+ T cells (where T cell 1 is stacked on T cell 2); as well as CD4-CD8+ T cells and CD4+CD8- T cells

Figure 31. *Schematic demonstrating the impact of tissue section thickness on marker detection and annotation in imaging mass cytometry*

Analysis factors. Expression levels of antibodies is only one of several factors leading to the final decision on annotation/identity of the cell clusters. Biological knowledge has a critical contribution, particularly in our setting. Thus, the use of low, mid and high expression is never undertaken in isolation; it is always used in conjunction with biologically expected change in a combination of markers. The calling of high and low expression is also restricted to proteins which are expected to vary for a biological reason, typically differentiation, plasticity or phenotypic subtyping. For example, monocytes are precursors of macrophages, and lose CD14 while gaining CD68 expression as they differentiate into macrophages. These reciprocal changes are observed in these cell types - $CD14^{lo}$ monocytes have high CD68 expression while those with high CD14 have low CD68 expression. Thus, the calling/annotation of $CD14^{lo}$ monocytes does not rely on CD14 expression alone but also on the <u>concomitant</u> and appropriate expression of other biologically related proteins, that have been well established biologically for that particular group of cells. This is also the case for KRT expression in aberrant basaloid intermediate cells where a collection of markers, KRT5, KRT17, pro-SpC, EPCAM and CD45 are evaluated together before a final call is made for the identity of a cell.
These data are found in Suppl Table 4 which shows expression of all relevant antibodies together for the biologically appropriate groups of cell types (eg differentiating monocyte-macrophage myeloid cells and differentiating alveolar epithelial cells).

Low, mid and high expression is done after consolidation of numerical rather than visually determined signals, aggregated from the average expression for all cells in any one cluster, in order to reduce the contribution of individual/single poor or erroneous staining. Multiple iterative 'loops' of analyses are performed to fine tune final conclusions on expression. In the following paragraphs, we summarise

how we have done this, in order to address the comment '…also analysis factors we do not encounter when analysing single cell data such as: the size of cells, the size of the nuclei, where they are cut, etc..'

A computational analytical algorithm (Deep Cell)[1] first segments the cells, identifying the nucleus as the centre of a cell, and using the cytoplasmic and cell surface markers to map out the rest of each cell. Segmentation is performed as shown in Figure R4A. Edges of unusually shaped cells (see below) that are captured without nucleus are discarded computationally. The average intensity of each marker for each cell is then obtained and submitted for unbiased dimensionality reduction and clustering process with R – Phenograph. All samples are integrated and batch correction is performed (Harmony) and, finally, an overall representation of the marker expression per cluster across all samples (expression density histogram) is generated(Figure R3B-C). High numbers of single cells (n= 313,000) help to reduce the contribution of signals due to technical variability; and consistent, computationally applied rules about which cells to keep and which to discard across all cells and all samples provide a final, unbiased single cell landscape.

A.

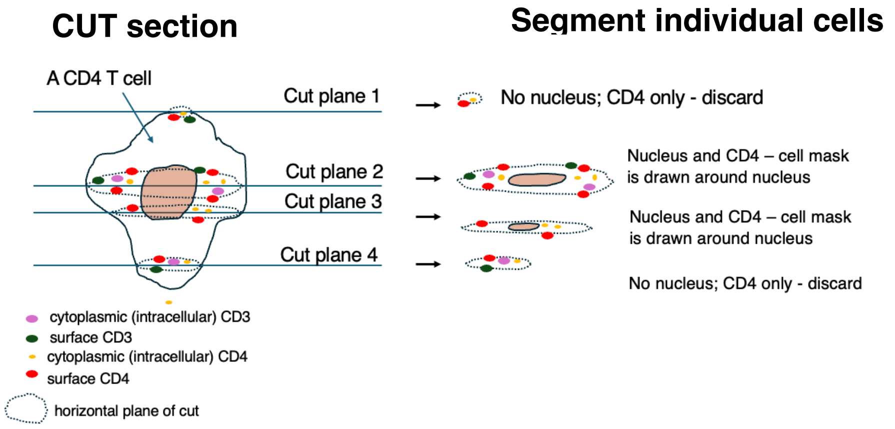

B.

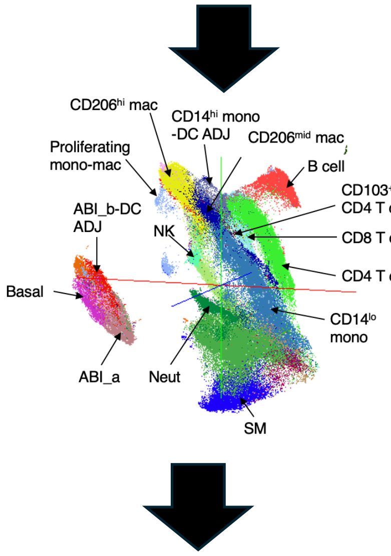

C.

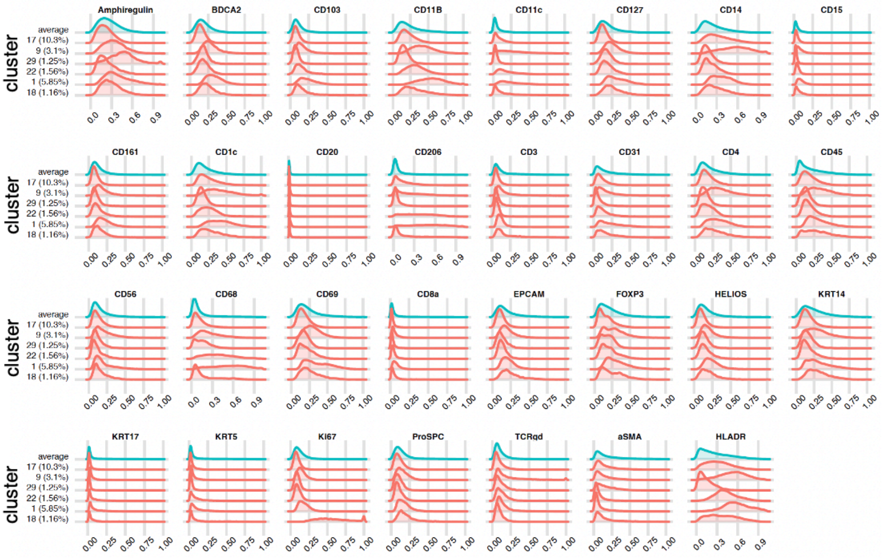

Figure R4. *A.Diagram demonstrating the process of single cell segmentation by DeepCell B-C. UMAP showing final annotated clusters and scaled marker expression density plots for selected cell clusters*

Once these steps have been completed, we proceed to annotation (Suppl Figure 1).

Extended description on role of different spatial analytical methods

The different methods we used add strength to each other and provide different information. Thus,

1. The pair-wise comparison provided by the cross-PCF (Fig 3B) identifies, in an unbiased way, the <u>pairs</u> of cell types that are spatially associated with each other more frequently than would occur by chance (i.e., under complete spatial randomness)
2. The triplet comparison provided by the NCF (Fig G-J) identifies, in an unbiased way, <u>triples</u> of cell types that are physically associated with each other
3. The neighbourhood analysis in Fig 4 brings together all pairs of cell types that are spatially co-located with our cell type of interest.

Relationship between ACN and CCN. Direct cell/cell analyses (CCN) used in Figure 5, we ask the following - of the pairs of cell types which are co-located within 20um of each other [ie g(r=20)>1 in the cross-PCF analyses], which cell pairs are also in direct physical contact? To provide greater clarity, please see the diagram below (Figure R5):

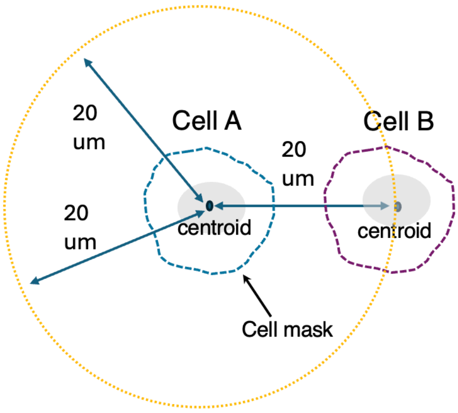

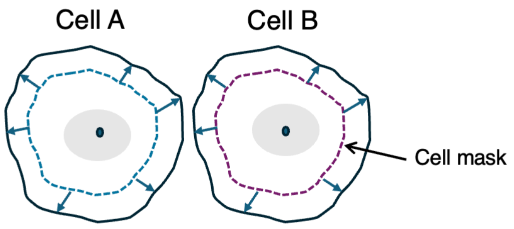

Cross PCF analysis asks if there is a greater number of cells of all types (in this case cells of type B) around a 20 μm radius from the centroid of Cell A [for g(r=20)]. Calculation of cross-PCF is performed using the cell centroids of a pair of cells. The 'cell centroid' is a point in the centre of of the nucleus of the cell.

ACN or adjacency cell network analysis asks if cell pairs that are found co-located with each other [g(r=20)>1] is also in physical contact with each other. To do this it takes the segmentation masks of all cells for these pairs of cell types, inflates the edge by a pre-determined 5μm and asks if their edges touch each other.

Figure R5. *Figures show how cross-PCF is related to ACN, and the additional information it provides. CCN (the final analysis used in Figure 5) is derived from ACN as follows - for each cell type pair identified as contacting in ACN (e.g., cell types A and B), the proportion of type A cells that is in contact with at least one of cell type B is calculated, and then the z-score. A p value is computed for the z-score associated with each pair of cell types. The data are then filtered to retain pairs of cell types with g(r=20)>1, positive z-scores and p < 0.05 to create a network in which each connection ('edge') between cell pairs encompasses these three calculations.*

ACN and CCN add value to the cross-PCF. The cross-PCF approach taken in Fig 3 considers cell centres only, ignoring cell shape. Advantages of this approach are the ability to compare the distribution of cell centres directly against a null distribution (complete spatial randomness) and its simplicity to compute. However, this approach has limitations. Two cell centres within short range (20um) may belong to cells which are not in physical contact. Additionally, cells with unusual (eg convoluted/dendritic) shapes may be in contact but their centroids may be more than 20um apart. ACN and CCN use the segmentation mask to ensure that the cells considered are in direct contact. We expect the two complementary methods to yield similar results, as the reviewer notes; by using both methods, rather than limiting our analysis to a single method, we can be more confident in our biological conclusions.

Extended information on segmentation of cells:

The Deep Cell segmentation method leverages deep learning models [mainly convolutional neural networks (CNNs)], to accurately identify and separate individual cells in microscopy images. One of the main challenges in cell segmentation is dealing with overlapping or closely packed cells, which can lead to under-segmentation (merging multiple cells as one) or over-segmentation (splitting one cell into several).

To prevent overlapping cells from being merged, Deep Cell segmentation typically includes these key strategies:

1. Instance segmentation models: Rather than simple semantic segmentation (classifying pixels as "cell" or "background"), Deep Cell uses instance-aware models like Mask R-CNN or U-Net with watershed post-processing. These models learn to distinguish individual cells, even when they are in close proximity or slightly overlapping.
2. Boundary-aware training: The model is trained on labeled datasets that emphasize clear cell boundaries. This includes using special loss functions that penalize boundary errors, and data augmentations that expose the model to various crowding scenarios.
3. Post-processing techniques: After the model predicts a cell probability map, methods like watershed transformation, distance transform, or contour detection are applied. These help split touching or slightly overlapping cells by finding their centers and propagating boundaries outward.
4. Probability maps or distance maps: Deep Cell often predicts not just a binary mask, but also distance-to-center maps or boundary probability maps. These help the model infer where one cell ends and another begins, even in densely packed regions.

By combining a robust deep neural network with biologically informed post-processing, Deep Cell segmentation reliably separates overlapping cells and ensures each is uniquely identified as an instance, which is essential for downstream quantitative analysis.

References

1. Greenwald, N. F. *et al.* Whole-cell segmentation of tissue images with human-level performance using large-scale data annotation and deep learning. *Nature Biotechnology* **40**, 555-565, doi:10.1038/s41587-021-01094-0 (2022).
2. Hunter, B. *et al.* OPTIMAL: An OPTimized Imaging Mass cytometry AnaLysis framework for benchmarking segmentation and data exploration. *Cytometry Part A* **105**, 36-53, doi:https://doi.org/10.1002/cyto.a.24803 (2024).
3. Chevrier, S. *et al.* Compensation of Signal Spillover in Suspension and Imaging Mass Cytometry. *Cell Systems* **6**, 612-620.e615, doi:10.1016/j.cels.2018.02.010 (2018).
4. Windhager, J. *et al.* An end-to-end workflow for multiplexed image processing and analysis. *Nature Protocols* **18**, 3565-3613, doi:10.1038/s41596-023-00881-0 (2023).
5. Korsunsky, I. *et al.* Fast, sensitive and accurate integration of single-cell data with Harmony. *Nat Methods* **16**, 1289-1296, doi:10.1038/s41592-019-0619-0 (2019).
6. Adams, T. S. *et al.* Single-cell RNA-seq reveals ectopic and aberrant lung-resident cell populations in idiopathic pulmonary fibrosis. *Sci Adv* **6**, eaba1983, doi:10.1126/sciadv.aba1983 (2020).
7. Habermann, A. C. *et al.* Single-cell RNA sequencing reveals profibrotic roles of distinct epithelial and mesenchymal lineages in pulmonary fibrosis. *Sci Adv* **6**, eaba1972, doi:10.1126/sciadv.aba1972 (2020).
8. Kathiriya, J. J. *et al.* Human alveolar type 2 epithelium transdifferentiates into metaplastic KRT5+ basal cells. *Nature Cell Biology* **24**, 10-23, doi:10.1038/s41556-021-00809-4 (2022).
