# Supplementary material for: Temporo-spatial cellular atlas of the regenerating alveolar niche in idiopathic pulmonary fibrosis
Source: Nat Commun. 2025 Aug 4;16:7150. doi: 10.1038/s41467-025-61880-1 (PMC12322046; doi:10.1038/s41467-025-61880-1)
Supplement: Supplementary file 2 — Reporting Summary [file 41467_2025_61880_MOESM2_ESM.pdf]

nature portfolio

Corresponding author(s): Prof Ling Pei Ho
Last updated by author(s): 25/06/25

Reporting Summary

Nature Portfolio wishes to improve the reproducibility of the work that we publish. This form provides structure for consistency and transparency in reporting. For further information on Nature Portfolio policies, see our Editorial Policies and the Editorial Policy Checklist.

Statistics

For all statistical analyses, confirm that the following items are present in the figure legend, table legend, main text, or Methods section.

n/a	Confirmed	
☐	☒	The exact sample size (n) for each experimental group/condition, given as a discrete number and unit of measurement
☐	☒	A statement on whether measurements were taken from distinct samples or whether the same sample was measured repeatedly
☐	☒	The statistical test(s) used AND whether they are one- or two-sided<br>*Only common tests should be described solely by name; describe more complex techniques in the Methods section.*
☐	☒	A description of all covariates tested
☐	☒	A description of any assumptions or corrections, such as tests of normality and adjustment for multiple comparisons
☐	☒	A full description of the statistical parameters including central tendency (e.g. means) or other basic estimates (e.g. regression coefficient) AND variation (e.g. standard deviation) or associated estimates of uncertainty (e.g. confidence intervals)
☐	☒	For null hypothesis testing, the test statistic (e.g. F, t, r) with confidence intervals, effect sizes, degrees of freedom and P value noted<br>*Give P values as exact values whenever suitable.*
☒	☐	For Bayesian analysis, information on the choice of priors and Markov chain Monte Carlo settings
☒	☐	For hierarchical and complex designs, identification of the appropriate level for tests and full reporting of outcomes
☐	☒	Estimates of effect sizes (e.g. Cohen's d, Pearson's r), indicating how they were calculated

Our web collection on statistics for biologists contains articles on many of the points above.

Software and code

Policy information about availability of computer code

Data collection	Imaging mass cytometry (IMC) data was acquired using the Hyperion imaging system (Standard BioTools) following manufacturer recommended procedure. The selected regions of interest were laser ablated at 200Hz, and data visualized using a commercial acquisition software (Hyperion imaging system v7.0.8493.0, Standard BioTools)
Data analysis	All IMC MCD files were inspected for the quality of staining (MCD viewer V. 1.0.560.2 Standard BioTools)<br>A customized pipeline SpOOx (Spatial Omics Oxford Pipeline) was used to process all data through MCD file conversion, segmentation, signal extraction, clustering and spatial analysis. All source code, methods and a tutorial are available at https://github.com/LingPeiHo/Ho_Taylor_Byrne-SpOOx-2.0<br>Visualization and final analysis of the data was achieved by Multi-Dimensional Viewer and all source code used are available at https://github.com/LingPeiHo/Ho_Taylor_Byrne-SpOOx-2.0<br>Tutorials and further documentation for the use of MDV are available on the web site: https://mdv.molbiol.ox.ac.uk/<br><br>Software<br><br>Name of software Version Source Identifier<br>imctools 2.1.8 https://github.com/BodenmillerGroup/imctools RRID:SCR_017132<br>Deepcell 0.12.4 https://vanvalen.github.io/about/ RRID:SCR_022197<br>Phenograph 1.5.2 https://github.com/JinmiaoChenLab/Rphenograph RRID:SCR_016919<br>Harmony 0.1.1 https://github.com/slowkow/harmonypy RRID:SCR_022206<br>Ruffus 2.6.3 http://www.ruffus.org.uk/ RRID:SCR_022196

QuPath 0.5.1 https://qupath.github.io/ https://doi.org/10.1038/s41598-017-17204-5
MCD 1.0.560.6 https://www.standardbio.com/products-services/software RRID:SCR_023007
Catalyst R 1.32.0 http://bioconductor.org/packages/CATALYST/ RRID:SCR_017127
diffcyt R package (version 1.8.8) 1.8.8 https://www.bioconductor.org/packages/release/bioc/html/diffcyt.html RRID:SCR_023006
Cellchat 1.6.1 https://github.com/jinworks/CellChat RRID:SCR_021946
Seurat 4.3 https://satijalab.org/seurat/ RRID:SCR_007322

For manuscripts utilizing custom algorithms or software that are central to the research but not yet described in published literature, software must be made available to editors and reviewers. We strongly encourage code deposition in a community repository (e.g. GitHub). See the Nature Portfolio guidelines for submitting code & software for further information.

Data

Policy information about availability of data

All manuscripts must include a data availability statement. This statement should provide the following information, where applicable:

- Accession codes, unique identifiers, or web links for publicly available datasets
- A description of any restrictions on data availability
- For clinical datasets or third party data, please ensure that the statement adheres to our policy

The spatial mass cytometry data files (MCD) generated in this study have been deposited in the Zenodo database under accession code 10930946 (https://doi.org/10.5281/zenodo.10930946). The processed source data used in the analyses presented in the paper is available for download as an online resource in Multi-Dimensional Viewer (MDV) accessible with the hyperlink, https://mdv.molbiol.ox.ac.uk/projects/mdv_project/7430. Specific source data for graphs are also provided in the source data file.

Research involving human participants, their data, or biological material

Policy information about studies with human participants or human data. See also policy information about sex, gender (identity/presentation), and sexual orientation and race, ethnicity and racism.

Reporting on sex and gender	Sex is reported in the patient information table (Supplementary table 1). Sex and gender were considered for study and reflects distribution of sex in the disease. Gender was self-reported.
Reporting on race, ethnicity, or other socially relevant groupings	Data on race and ethnicity and other social groupings were not used as variables in this study and hence were not obtained
Population characteristics	The characteristics of the patients from which samples were obtained presented in supplementary table 1
Recruitment	Diseased lung tissue was obtained from patients with a diagnosis of Idiopathic pulmonary Fibrosis (IPF) based on standard diagnostic criteria and multi-disciplinary team (MDT) consensus, at the point of lung transplantation at the Institute of Transplantation, Newcastle Upon Tyne Hospitals NHS Foundation Trust.
Ethics oversight	All patients provided informed consent for use of their tissue via the Cellular and Molecular Mechanisms in Chronic Lung Diseases (EXPLANT) study which was approved by the NHS Research Ethics Service (11/NE/0291) and was sponsored by Newcastle Upon Tyne Hospitals NHS Foundation Trust (R&D ref 5885).

Note that full information on the approval of the study protocol must also be provided in the manuscript.

Field-specific reporting

Please select the one below that is the best fit for your research. If you are not sure, read the appropriate sections before making your selection.

[x] Life sciences [ ] Behavioural & social sciences [ ] Ecological, evolutionary & environmental sciences

For a reference copy of the document with all sections, see nature.com/documents/nr-reporting-summary-flat.pdf

Life sciences study design

All studies must disclose on these points even when the disclosure is negative.

Sample size	A statistical method was not used for sample size determination
Data exclusions	None
Replication	Key findings were replicated by another technology (Immunoflouorescence)
Randomization	The order of data acquisition on the Hyperion imaging system was randomized
Blinding	All data acquisition was conducted blinded to associated clinical data

Reporting for specific materials, systems and methods

We require information from authors about some types of materials, experimental systems and methods used in many studies. Here, indicate whether each material, system or method listed is relevant to your study. If you are not sure if a list item applies to your research, read the appropriate section before selecting a response.

Materials & experimental systems

n/a	Involved in the study
☐	☒ Antibodies
☐	☐ Eukaryotic cell lines
☐	☐ Palaeontology and archaeology
☐	☐ Animals and other organisms
☐	☒ Clinical data
☐	☐ Dual use research of concern
☐	☐ Plants

Methods

n/a	Involved in the study
☐	☐ ChIP-seq
☐	☐ Flow cytometry
☐	☐ MRI-based neuroimaging

Antibodies

Antibodies used

Detailed data on antibodies provided in supplementary table 5 and are also presented below in this reporting summary

Imaging mass cytometry antibodies

Target Clone Supplier Cat number Lot Metal Concentration

CD45 D9M8I Cell Signal Technology 13917BF 11 141 5
CD68 KP1 Biolegend 916104 B283618 142 5
CD8a C8/144B Biolegend 372902 B298974 143 6
Ki67 Polyclonal Novus NB500-170 H-2 144 5
alpha-Smooth Muscle Actin 1A4/asm-1 Novus NBP2-33006 IBR082005B 145 2
FoxP3 236A/E7 Abcam ab20034 GR3443434-2 147 20
CD11c 2F1C10 Protein Tech 60258-1-Ig 10002357 149 2
CD103 EPR22590-27 Abcam ab254201 GR3288218 152 5
CD56 E7X9M Cell Signal Technology 99746 2 153 20
Helios E4L5U Cell Signal Technology 89270 2 154 10
CD69 15B5G2 Cell Signal Technology NBP2-25236 0320453162-03 155 2
Epcam Polyclonal Abcam ab71916 CR3357742-1 156 0.5
CD20 4A7G3 Protein Tech 60271-1-Ig 1003534 158 0.5
CD206 2A6A10 Protein Tech 60143-1-Ig 10004170 159 0.5
CD127 EPR2955(2) Abcam ab240225 GR3375511-1 160 1
KRT7/17 C-46 Biolegend 628702 B254700 161 5
CD1c 2A7C11 Novus NBP2-61726 170831 162 2
CD15 SSEA-1 Biolegend 323002 B254011 163 3
KRT14 2G1E2 Protein Tech 60320-1-Ig 1332030 164 2
TCRδ H-41 Santa Cruz Biotechnology 91921 sc-100289 165 10
Amphiregulin 1A1G9 Protein Tech 66433-1-IG 10004459 166 1.5
CD11B 1C7C2 Protein Tech 66519-1-Ig 10003969 167 0.5
BDCA2 10E6.1 Millipore MABF94 3698182 168 20
KRT5 2C2 Thermofisher MA5-17057 VJ3094812 169 5
CD3 PC Sera Fluidigm 3170019-D 2101806-28 170 6
ProSPC Polyclonal Novus NPB1-60117 KL63276-170622 171 5
CD31 EPR3094 Abcam ab207090 G-R3229164-12 172 3
CD161 OTI1D8 Abcam ab273666 GR33527516 173 8
CD4 EPR6855 Abcam Ab181724 GR3285644-12 174 4
HLA-DR LN3 Biolegend 327002 B262251 175 1.5
CD14 EPR3653 Abcam EPR3653 GR33451137 176 1

Immunoflouorescence antibodies

Target Clone Supplier Cat no. Conjugate Working Concentration

Cytokeratin 17 EP1623 Abcam AB185032 Alexa Fluor® 488 1 in 200
CD15/SSEA1 MC480 Cell Signaling Technology #4744 NA 1 in 200
Prosurfactant Protein C Rabbit polyclonal Abcam AB90716 NA 1 in 400
Cytokeratin 5 EP1601Y Abcam AB193895 Alexa Fluor® 647 1 in 200

MRC-1 CL0387 Atlas antibodies AMAB90746 NA 1 in 500
BDCA-2 10E6.1 Merck Millipore MABF94 NA 1 in 100
Fibronectin F1 Abcam AB198934 Alexa Fluor® 647 1 in 200
Goat anti Rabbit IgG Polyclonal Thermo Fischer Scientific A21039 Alexa Fluor® 750 1 in 300
Goat anti-mouse IgG2b Polyclonal Thermo Fischer Scientific A21144 Alexa Fluor® 568 1 in 300
ProSPC H-8 Santa Cruz Biotechnology sc-518029 NA 1 in 200
ki67 MIB-1 Agilent Dako GA62661-2 NA 1 in 100

Validation

Antibody clones were validated and optimized using two-colour immune-fluorescence staining of appropriate positive control and lung tissue. Here, slides were imaged using a Axio Scan Z1 fluroscence microscope (Zeiss). Using Zeiss software, the microscope was configured for AF405, AF488, AF568, AF647, and AF750, and the entire area of the tissue section is selected using the software for higher resolution scanning, utilizing a plan apochromat 40x 0.95 korr M27 objective. Images were saved on a computer for further processing using custom Fiji/Image J macros.
Following verification of staining pattern and performance quality, approved antibodies were subject to lanthanide metal conjugation using a Maxpar X8 metal conjugation kit following manufacturer's protocol (Standard Biotools). Successful metal conjugation was verified by binding the antibody to iridium labelled antibody capture beads AbC™ Total Antibody Compensation Beads (Thermo Fisher) and acquiring on a Helios system (Standard Bio-tools).

Eukaryotic cell lines

Policy information about cell lines and Sex and Gender in Research

Cell line source(s)

State the source of each cell line used and the sex of all primary cell lines and cells derived from human participants or vertebrate models.

Authentication

Describe the authentication procedures for each cell line used OR declare that none of the cell lines used were authenticated.

Mycoplasma contamination

Confirm that all cell lines tested negative for mycoplasma contamination OR describe the results of the testing for mycoplasma contamination OR declare that the cell lines were not tested for mycoplasma contamination.

Commonly misidentified lines
(See ICLAC register)

Name any commonly misidentified cell lines used in the study and provide a rationale for their use.

Palaeontology and Archaeology

Specimen provenance

Provide provenance information for specimens and describe permits that were obtained for the work (including the name of the issuing authority, the date of issue, and any identifying information). Permits should encompass collection and, where applicable, export.

Specimen deposition

Indicate where the specimens have been deposited to permit free access by other researchers.

Dating methods

If new dates are provided, describe how they were obtained (e.g. collection, storage, sample pretreatment and measurement), where they were obtained (i.e. lab name), the calibration program and the protocol for quality assurance OR state that no new dates are provided.

☐ Tick this box to confirm that the raw and calibrated dates are available in the paper or in Supplementary Information.

Ethics oversight

Identify the organization(s) that approved or provided guidance on the study protocol, OR state that no ethical approval or guidance was required and explain why not.

Note that full information on the approval of the study protocol must also be provided in the manuscript.

Animals and other research organisms

Policy information about studies involving animals; ARRIVE guidelines recommended for reporting animal research, and Sex and Gender in Research

Laboratory animals

For laboratory animals, report species, strain and age OR state that the study did not involve laboratory animals.

Wild animals

Provide details on animals observed in or captured in the field; report species and age where possible. Describe how animals were caught and transported and what happened to captive animals after the study (if killed, explain why and describe method; if released, say where and when) OR state that the study did not involve wild animals.

Reporting on sex

Indicate if findings apply to only one sex; describe whether sex was considered in study design, methods used for assigning sex. Provide data disaggregated for sex where this information has been collected in the source data as appropriate; provide overall numbers in this Reporting Summary. Please state if this information has not been collected. Report sex-based analyses where performed, justify reasons for lack of sex-based analysis.

Field-collected samples	*For laboratory work with field-collected samples, describe all relevant parameters such as housing, maintenance, temperature, photoperiod and end-of-experiment protocol OR state that the study did not involve samples collected from the field.*
Ethics oversight	*Identify the organization(s) that approved or provided guidance on the study protocol, OR state that no ethical approval or guidance was required and explain why not.*

Note that full information on the approval of the study protocol must also be provided in the manuscript.

Clinical data

Policy information about clinical studies

All manuscripts should comply with the ICMJEguidelines for publication of clinical research and a completedCONSORT checklist must be included with all submissions.

Clinical trial registration	This study was not part of a clinical trial
Study protocol	This study was not part of a clinical trial, hence not applicable
Data collection	Data was collected by perusal of the clinical and pathological records of the included patient samples
Outcomes	No primary or secondary outcome was defined

Dual use research of concern

Policy information about dual use research of concern

Hazards

Could the accidental, deliberate or reckless misuse of agents or technologies generated in the work, or the application of information presented in the manuscript, pose a threat to:

No	Yes	
☐	☐	Public health
☐	☐	National security
☐	☐	Crops and/or livestock
☐	☐	Ecosystems
☐	☐	Any other significant area

Experiments of concern

Does the work involve any of these experiments of concern:

No	Yes	
☐	☐	Demonstrate how to render a vaccine ineffective
☐	☐	Confer resistance to therapeutically useful antibiotics or antiviral agents
☐	☐	Enhance the virulence of a pathogen or render a nonpathogen virulent
☐	☐	Increase transmissibility of a pathogen
☐	☐	Alter the host range of a pathogen
☐	☐	Enable evasion of diagnostic/detection modalities
☐	☐	Enable the weaponization of a biological agent or toxin
☐	☐	Any other potentially harmful combination of experiments and agents

Plants

Seed stocks | *Report on the source of all seed stocks or other plant material used. If applicable, state the seed stock centre and catalogue number. If plant specimens were collected from the field, describe the collection location, date and sampling procedures.*

Novel plant genotypes | *Describe the methods by which all novel plant genotypes were produced. This includes those generated by transgenic approaches, gene editing, chemical/radiation-based mutagenesis and hybridization. For transgenic lines, describe the transformation method, the number of independent lines analyzed and the generation upon which experiments were performed. For gene-edited lines, describe the editor used, the endogenous sequence targeted for editing, the targeting guide RNA sequence (if applicable) and how the editor was applied.*

Authentication | *Describe any authentication procedures for each seed stock used or novel genotype generated. Describe any experiments used to assess the effect of a mutation and, where applicable, how potential secondary effects (e.g. second site T-DNA insertions, mosiacism, off-target gene editing) were examined.*

ChIP-seq

Data deposition

☐ Confirm that both raw and final processed data have been deposited in a public database such as GEO.

☐ Confirm that you have deposited or provided access to graph files (e.g. BED files) for the called peaks.

Data access links
May remain private before publication. | *For "Initial submission" or "Revised version" documents, provide reviewer access links. For your "Final submission" document, provide a link to the deposited data.*

Files in database submission | *Provide a list of all files available in the database submission.*

Genome browser session
(e.g. UCSC) | *Provide a link to an anonymized genome browser session for "Initial submission" and "Revised version" documents only, to enable peer review. Write "no longer applicable" for "Final submission" documents.*

Methodology

Replicates | *Describe the experimental replicates, specifying number, type and replicate agreement.*

Sequencing depth | *Describe the sequencing depth for each experiment, providing the total number of reads, uniquely mapped reads, length of reads and whether they were paired- or single-end.*

Antibodies | *Describe the antibodies used for the ChIP-seq experiments; as applicable, provide supplier name, catalog number, clone name, and lot number.*

Peak calling parameters | *Specify the command line program and parameters used for read mapping and peak calling, including the ChIP, control and index files used.*

Data quality | *Describe the methods used to ensure data quality in full detail, including how many peaks are at FDR 5% and above 5-fold enrichment.*

Software | *Describe the software used to collect and analyze the ChIP-seq data. For custom code that has been deposited into a community repository, provide accession details.*

Flow Cytometry

Plots

Confirm that:

☐ The axis labels state the marker and fluorochrome used (e.g. CD4-FITC).

☐ The axis scales are clearly visible. Include numbers along axes only for bottom left plot of group (a 'group' is an analysis of identical markers).

☐ All plots are contour plots with outliers or pseudocolor plots.

☐ A numerical value for number of cells or percentage (with statistics) is provided.

Methodology

Sample preparation | *Describe the sample preparation, detailing the biological source of the cells and any tissue processing steps used.*

Instrument | *Identify the instrument used for data collection, specifying make and model number.*

Software | *Describe the software used to collect and analyze the flow cytometry data. For custom code that has been deposited into a community repository, provide accession details.*

Cell population abundance | *Describe the abundance of the relevant cell populations within post-sort fractions, providing details on the purity of the samples and how it was determined.*

Gating strategy | *Describe the gating strategy used for all relevant experiments, specifying the preliminary FSC/SSC gates of the starting cell population, indicating where boundaries between "positive" and "negative" staining cell populations are defined.*

☐ Tick this box to confirm that a figure exemplifying the gating strategy is provided in the Supplementary Information.

Magnetic resonance imaging

Experimental design

Design type | *Indicate task or resting state; event-related or block design.*

Design specifications | *Specify the number of blocks, trials or experimental units per session and/or subject, and specify the length of each trial or block (if trials are blocked) and interval between trials.*

Behavioral performance measures | *State number and/or type of variables recorded (e.g. correct button press, response time) and what statistics were used to establish that the subjects were performing the task as expected (e.g. mean, range, and/or standard deviation across subjects).*

Acquisition

Imaging type(s) | *Specify: functional, structural, diffusion, perfusion.*

Field strength | *Specify in Tesla*

Sequence & imaging parameters | *Specify the pulse sequence type (gradient echo, spin echo, etc.), imaging type (EPI, spiral, etc.), field of view, matrix size, slice thickness, orientation and TE/TR/flip angle.*

Area of acquisition | *State whether a whole brain scan was used OR define the area of acquisition, describing how the region was determined.*

Diffusion MRI ☐ Used ☐ Not used

Preprocessing

Preprocessing software | *Provide detail on software version and revision number and on specific parameters (model/functions, brain extraction, segmentation, smoothing kernel size, etc.).*

Normalization | *If data were normalized/standardized, describe the approach(es): specify linear or non-linear and define image types used for transformation OR indicate that data were not normalized and explain rationale for lack of normalization.*

Normalization template | *Describe the template used for normalization/transformation, specifying subject space or group standardized space (e.g. original Talairach, MNI305, ICBM152) OR indicate that the data were not normalized.*

Noise and artifact removal | *Describe your procedure(s) for artifact and structured noise removal, specifying motion parameters, tissue signals and physiological signals (heart rate, respiration).*

Volume censoring | *Define your software and/or method and criteria for volume censoring, and state the extent of such censoring.*

Statistical modeling & inference

Model type and settings | *Specify type (mass univariate, multivariate, RSA, predictive, etc.) and describe essential details of the model at the first and second levels (e.g. fixed, random or mixed effects; drift or auto-correlation).*

Effect(s) tested | *Define precise effect in terms of the task or stimulus conditions instead of psychological concepts and indicate whether ANOVA or factorial designs were used.*

Specify type of analysis: ☐ Whole brain ☐ ROI-based ☐ Both

Statistic type for inference (See Eklund et al. 2016) | *Specify voxel-wise or cluster-wise and report all relevant parameters for cluster-wise methods.*

Correction | *Describe the type of correction and how it is obtained for multiple comparisons (e.g. FWE, FDR, permutation or Monte Carlo).*

Models & analysis

n/a | Involved in the study
- [ ] [ ] Functional and/or effective connectivity
- [ ] [ ] Graph analysis
- [ ] [ ] Multivariate modeling or predictive analysis

Functional and/or effective connectivity	*Report the measures of dependence used and the model details (e.g. Pearson correlation, partial correlation, mutual information).*
Graph analysis	*Report the dependent variable and connectivity measure, specifying weighted graph or binarized graph, subject- or group-level, and the global and/or node summaries used (e.g. clustering coefficient, efficiency, etc.).*
Multivariate modeling and predictive analysis	*Specify independent variables, features extraction and dimension reduction, model, training and evaluation metrics.*
